# Introducing a new 7-ring fused diindenone-dithieno[3,2-*b*:2',3'-*d*]thiophene unit as a promising component for organic semiconductor materials

**DOI:** 10.3762/bjoc.18.94

**Published:** 2022-08-01

**Authors:** Valentin H K Fell, Joseph Cameron, Alexander L Kanibolotsky, Eman J Hussien, Peter J Skabara

**Affiliations:** 1 WestCHEM, School of Chemistry, University of Glasgow, Joseph Black Building, University Avenue, Glasgow, G12 8QQ, Scotland; 2 Institute of Physical-Organic Chemistry and Coal Chemistry, 02160 Kyiv, Ukraine

**Keywords:** dithienothiophene (DTT), fused ring system, organic field-effect transistor (OFET), organic semiconductor, thienoacene

## Abstract

A novel π-conjugated molecule, **EtH-T-DI-DTT** is reported, which is fused, rigid, and planar, featuring the electron-rich dithieno[3,2-*b*:2’,3’-*d*]thiophene (DTT) unit in the core of the structure. Adjacent to the electron-donating DTT core, there are indenone units with electron-withdrawing keto groups. To enable solubility in common organic solvents, the fused system is flanked by ethylhexylthiophene groups. The material is a dark, amorphous solid with an onset of absorption at 638 nm in CH_2_Cl_2_ solution, which corresponds to an optical gap of 1.94 eV. In films, the absorption onset wavelength is at 701 nm, which corresponds to 1.77 eV. An ionisation energy of 5.5 eV and an electron affinity of 3.3 eV were estimated by cyclic voltammetry measurements. We have applied this new molecule in organic field effect transistors. The material exhibited a p-type mobility up to 1.33 × 10^−4^ cm^2^ V^−1^ s^−1^.

## Introduction

In recent years, organic molecules with several fused aromatic rings have gained much attention. Fusing aromatic rings leads to planar structures, which extends the degree of π-conjugation [1]. In this way, the HOMO–LUMO gap can be narrowed [2]. Low HOMO–LUMO gaps are desirable for organic solar cells as the maximum photoflux density of the sun is at ca. 700 nm, corresponding to 1.77 eV [3]. However, fused systems have the drawback of being prone to poor solubility as a consequence of strong π–π interactions between the planar molecules [4]. Thus, attaching solubilising alkyl chains is necessary [5]. A common way to further decrease the HOMO–LUMO gap is attaching electron-donating and electron-accepting groups. Electron-rich units raise the *E*_HOMO_ of the molecule closer to vacuum level, whilst electron-withdrawing units lower the *E*_LUMO_ away from vacuum, leading to smaller HOMO–LUMO gaps [3].

A central aspect of the development of modern technology is the improvement of semiconductors. Semiconductors are, for example, applied in transistors (the basic unit of processors) [6], solar cells, and LEDs [7]. Inorganic compounds, e.g. III-V type inorganics are widely used, however, in recent years, organic molecules with semiconducting and fluorescent properties have emerged as an alternative with advantages such as solution processing [8–10] and ease of tunability of properties [11]. Although their thermal stability is lower than their inorganic counterparts [12], their properties, e.g. the HOMO–LUMO gap, can be tailored and fine-tuned by molecular design [3]. Depending on their structure and/or functional groups, they can be designed for a certain application, for example where charge transport is more important than photoluminescence quantum yield or *vice versa*. Introducing alkyl chains can provide solubility, enabling facile solution processing, such as device printing techniques [13]. Hence, there is an ongoing interest in structure–property relationships. The basic structural reason for semiconductivity in an organic molecule is usually an extended conjugated π-electron system [14]. Depending on how extended the system is, the HOMO–LUMO gap can be small enough for semiconductivity. However, conjugation can be interrupted if moieties within a π-electron system are twisted with respect to each other, preventing efficient overlap of p-orbitals of adjacent carbon atoms [15]. To prevent that, there is a large interest in creating rigid, planar molecules with low or no rotational degrees of freedom. This can be achieved by fusing π-conjugated ring structures [15]. In the solid state, fused systems are prone to form highly ordered π–π-stacked structures [16], leading to better bulk charge transport [17].

Another important aspect is solubility, which is generally poor in larger conjugated molecules [3]. This is even aggravated for fused molecules as increased rigidity leads to reduced solubility [4]. This is not only a problem for both purification and characterisation, but also for device fabrication since good solubility enables facile, energy-efficient solution processing [18], e.g. spin-coating. By introducing alkyl chains, solubility in organic solvents can be achieved [5]. Bulky side chains are more efficient in increasing solubility in comparison to linear alkyl chains, however, bulky or branched side chains also hinder the formation of π–π-stacks. Another problem about alkyl chains is their insulating nature, having an adverse effect on the charge mobilities [3].

**Figure 1:**
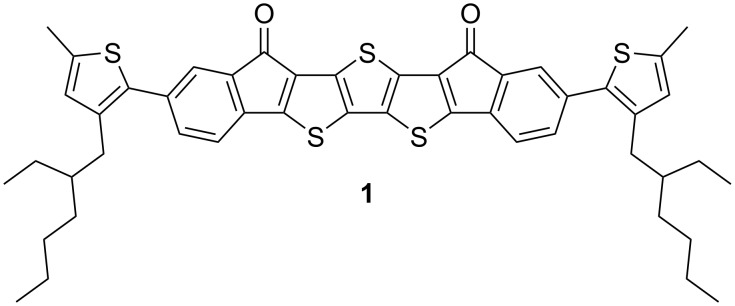
**EtH-T-DI-DTT** (**1**).

Here, we report a novel conjugated molecule, **EtH-T-DI-DTT** (**1**, Figure 1), which is fused, rigid, and planar, having an electron-rich [19] dithieno[3,2-*b*:2’,3’-*d*]thiophene (DTT) motif and electron-withdrawing [20] indenone moieties to reduce the HOMO–LUMO gap due to the donor–acceptor interaction [21].

With this strategy, fusing those systems should lead to greater π-delocalisation [15,22]. Moreover, the fused core system is flanked by thiophene groups with ethylhexyl groups which impart solubility [5] in common organic solvents such as tetrahydrofuran, chloroform or dichloromethane.

**Figure 2:**
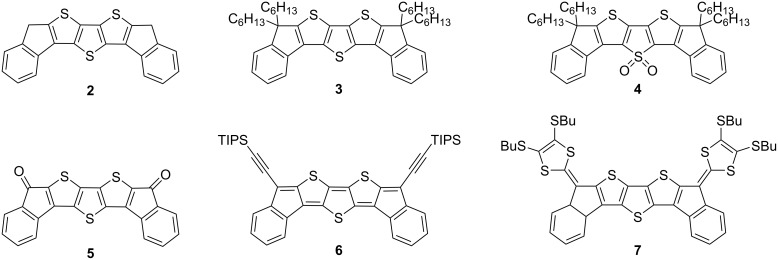
Previously published, ‘bent’ diindenodithienothiophenes [16,24–25].

There are numerous examples of fused, conjugated materials containing a thiophene motif. In a comprehensive and detailed review, Ozturk et al. summarised the chemistry and properties of fused thiophene systems [23], and pointed out their importance in the field of organic semiconductors. Earlier, we published a series of ‘bent’ diindenodithienothiophene derivatives (**2**–**4**, Figure 2) [16]. It was observed that oxidising the central sulfur atom significantly increased the solution photoluminescence quantum yield (PLQY) from 0.004 (**3**) to 0.72 (**4**).

The diketo derivative **5** of compound **2** has been further functionalised with (triisopropylsilyl)ethynyl [24] (**6**) or with 1,3-dithiole units [25] (**7**) by other research groups. The (triisopropylsilyl)ethynyl (TIPSE) groups are introduced to improve the solubility and solid-state order, fostering intermolecular π-orbital interactions [26]. Moreover, compound **6** features a quinoidal antiaromatic [10] structure. Antiaromaticity is reported to further decrease the energy gap. The electron-rich dithiole units provide an extended tetrathiafulvalene structure, leading to compound **7** exhibiting two reversible one-electron oxidations [25].

**Figure 3:**
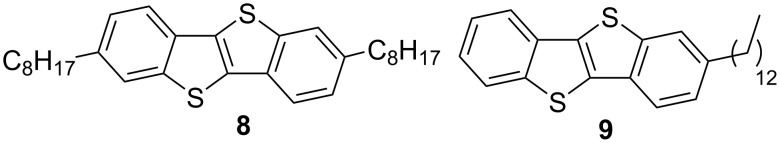
With crystalline films of 2,7-dioctyl[1]benzothieno[3,2-*b*][1]-benzothiophene (**8**), obtained by off-centre spin-coating, Bao et al. could obtain remarkable OFET hole mobilities of up to 43 cm^2^ V^−1^ s^−1^ [27]. An asymmetric analogue, which is only alkylated on one side (**9**), achieved OFET hole mobilities up to 17.22 cm^2^ V^−1^ s^−1^ in polycrystalline films obtained by thermal evaporation [28]; both examples prove the potential of thienoacenes in OFETs.

Fused thiophenes have been applied in various different molecules, exhibiting outstanding performances in certain applications. The highest hole mobility for organic semiconductors was achieved for thin, crystalline films of 2,7-dioctyl[1]benzothieno[3,2-*b*][1]-benzothiophene (**8**), shown in Figure 3, achieving a maximum hole mobility of 43 cm^2^ V^−1^ s^−1^, with an average of 25 cm^2^ V^−1^ s^−1^ [27].

The films were processed by a technique called off-centre spin-coating, in which the substrate is placed off the centre of the spin-coater. This leads to roughly unidirectional centrifugal forces in the substrate. The obtained crystallites had sizes of ca. 100 nm, while the crystallites in films obtained by on-centre spin-coating had smaller sizes of ca. 20 nm. The same core alkylated on only one side resulted in the asymmetric 2-tridecyl[1]benzothieno[3,2-*b*][1]-benzothiophene (**9**) [28]. In polycrystalline films obtained by thermal evaporation, average mobilities of 14.20 ± 2.55 cm^2^ V^−1^ s^−1^, with a maximum value of 17.2 cm^2^ V^−1^ s^−1^ were achieved.

**Figure 4:**
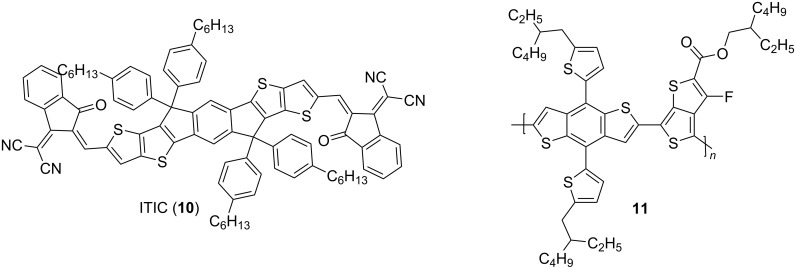
ITIC, a system with fused thiophenes, in combination with donor polymer **11**, also featuring a fused thiophene system, showed a remarkable PCE of 6.80%, the then best value for non-fullerene acceptor organic solar cells [17].

In recent years, fused thiophene molecules also achieved outstanding performances in OPVs. This was especially driven by recent developments in non-fullerene acceptors (NFA) [29]. One prominent example, ITIC (**10**), is shown in Figure 4. ITIC [17], in combination with polymer **11**, achieved a power conversion efficiency (PCE) of 6.8%, which was the best value for NFA organic solar cells at the time of publication.

**Figure 5:**
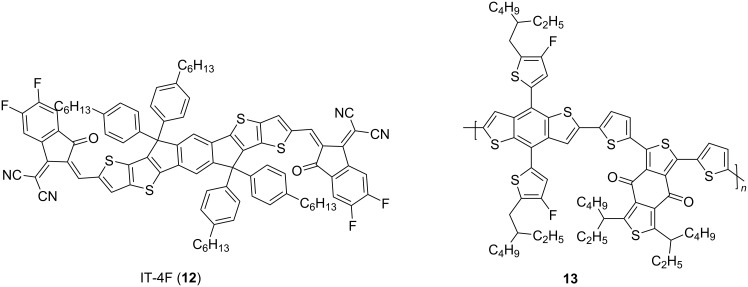
The fluorinated derivative of ITIC, IT-4F, achieved, with donor polymer **13**, PCEs in OPVs up to 17% [8].

Fluorination of ITIC, obtaining IT-4F (**12**), shown in Figure 5, reduced *E*_LUMO_. Combined with the donor polymer **13**, PCEs up to 17% were achieved [8].

**Figure 6:**
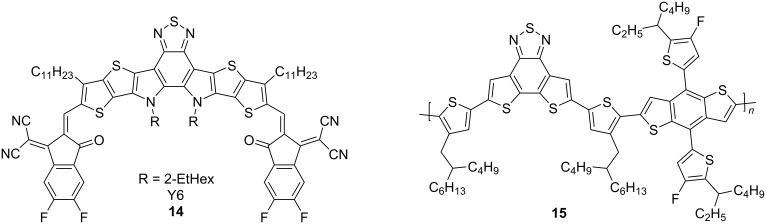
The non-fullerene acceptor Y6 (**14**) [30], in combination with donor polymer **15**, both fused thiophene systems, achieved a PCE of 18%, as published in 2019, the highest value up to then [31].

More recently, another fused-thiophene containing NFA was published, Y6 (**14**), shown in Figure 6 [30]. Y6 held the record for the highest value of an OPV with PCEs up to 18% upon blending with polymer **15** [31].

**Figure 7:**
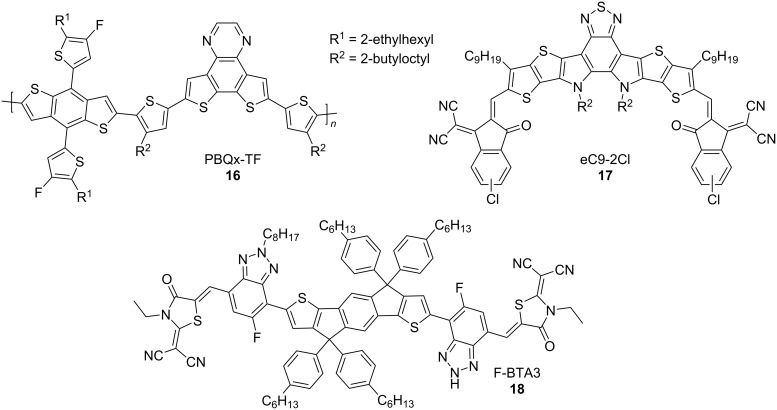
With a three component system of PBQx-TF, eC9-2Cl, and F-BTA3, a PCE of 19% was achieved [32].

This has now been surpassed; in 2021, Hou et al. reported a ternary OPV, using a mixture of the novel PBQx-TF (**16**) donor polymer and the non-fullerene acceptor eC9-2Cl (**17**), shown in Figure 7. In addition, a third material, F-BTA3 (**18**) was blended in [32]; all three materials are fused thiophene systems. The resulting OPV achieved a PCE of 19%.

## Results and Discussion

### Synthesis

**Scheme 1:**
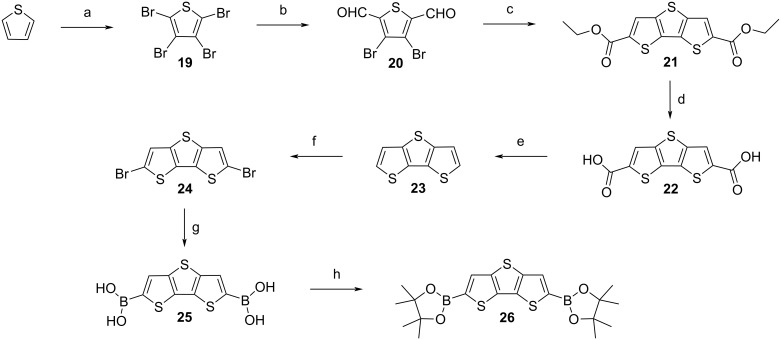
Synthetic route from thiophene to 2,6-bis(4,4,5,5-tetramethyl-1,3,2-dioxaborolan-2-yl)dithieno[3,2-*b*:2’,3’-*d*]thiophene (**26**): a) Br_2_, CHCl_3_, rt, overnight, reflux, 4 h, 94% [33]; b) *n*-BuLi, −78 °C, 30 min, 1-formylpiperidine, anhydrous THF, −78 °C, then rt, overnight [15], 88%; c) ethyl thioglycolate, anhydrous potassium carbonate, anhydrous *N,N*-dimethylformamide, rt, 3 d, 83% [15]; d) 1 M aqueous lithium hydroxide, THF, 4 h, 94% [15]; e) copper powder, quinoline, 230 °C, 1 h, 81% [15]; f) *N*-bromosuccinimide, CHCl_3_/glacial acetic acid, 0 °C, 1 h, rt, 1.5 h, 94% [15] ; g) *n*-BuLi, −90 °C, 20 min, triisopropyl borate, −80 °C, anhydrous THF, rt, overnight, 97% [34]; h) pinacol, toluene, 115 °C, 21.5 h, 79% [35].

The multi-step-synthesis of **EtH-T-DI-DTT** (**1**) begins from commercially available thiophene, which is used to synthesise 2,6-dibromodithienothiophene (**24**), according to previously published procedures [15,33], as shown in Scheme 1.

In a manner analogous to [34], dibromodithienothiophene **24** is lithiated with *n*-butyllithium at −90 °C, and the resulting species is reacted *in situ* with triisopropyl borate. After aqueous workup, dithieno[3,2-*b*:2’,3’-*d*]thiophene-2,6-diylboronic acid (**25**) is obtained, enabling subsequent Suzuki–Miyaura cross-coupling [36]. This palladium-catalysed cross coupling is preferred over a Stille cross-coupling due to the high toxicity of organotin reagents [37]. Moreover, purification of compound **25** is facile since it can be used for further reactions after re-precipitation in petroleum ether. In a manner similar to [35], it is possible to convert **25** into the corresponding pinacol ester, 2,6-bis(4,4,5,5-tetramethyl-1,3,2-dioxaborolan-2-yl)dithieno[3,2-*b*:2’,3’-*d*]thiophene (**26**) by stirring **25** with pinacol in refluxing toluene, but this has no beneficial impact on the subsequent cross-coupling. Compound **26** has been published previously by other groups [38–39], however, we here use a different protocol.

**Scheme 2:**
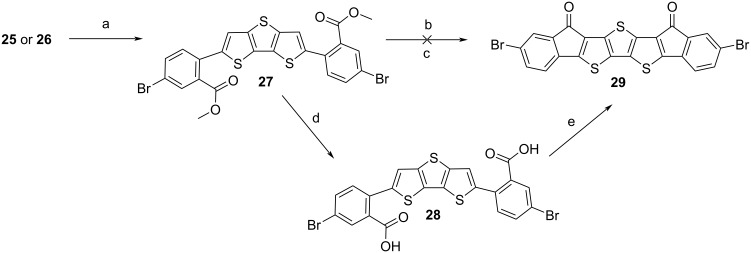
Ring closure of key intermediate **27** to achieve **29**: a) Methyl 5-bromo-2-iodobenzoate, Aliquat 336®, Pd(PPh_3_)_4_, K_2_CO_3_, THF/H_2_O, 70 °C, 40 h, 28% [40–41]; b) polyphosphoric acid [16], 100 °C, 4.5 h, then 130 °C, overnight, 0%; c) sulfuric acid, 115 °C, 6 h, 0% [42]; d) LiOH, THF, H_2_O, 70 °C, 24 h, 97% [15]; e) oxalyl chloride, dimethylformamide (cat.), anhydrous dichloromethane, rt, 30 min, then 50 °C, 3 h, AlCl_3_, anhydrous dichloromethane, 0 °C, 15 min, rt, 15 min, 40 °C, 13 h, 71% [24,43].

Intermediates **25** or **26** were reacted in Suzuki–Miyaura couplings [36] with commercially available methyl 5-bromo-2-iodobenzoate [40], to obtain the key intermediate dimethyl 6,6’-(dithieno[3,2-*b*:2’,3’-*d*]thiophene-2,6-diyl)bis(3-bromobenzoate) (**27**), which is a yellow solid (Scheme 2).

This step was found to be problematic. Purification was difficult, moreover, the batch-to-batch yield strongly fluctuated and was generally low. Intermediate **27** degraded during column chromatography, but this could be prevented by adding a small amount of triethylamine to the eluent [44]. Also, using a solvent mixture with a low polarity, which is necessary to receive a good separation from the side products, led to a precipitation of the compound on the column, and many attempts were necessary to find an ideal solvent mixture. Additionally, the reaction was very sensitive to changes in concentration, equivalents, amounts of reagents, and temperature. Running the reaction under anhydrous conditions, either conventionally [45] or in the microwave [46] did not improve the outcome. The optimised reaction and purification parameters can be found in Supporting Information File 1. According to the literature [41], Aliquat 336® can be added to Suzuki–Miyaura reactions. Here, this did not improve the yield, but decreased its fluctuation from batch to batch.

After sufficient amounts of intermediate **27** were isolated, attempts for ring-closure were made. Initial attempts using polyphosphoric acid [16] and sulfuric acid failed [42], therefore we tried Friedel–Crafts acylation. For that, **27** was hydrolysed to the corresponding diacid **28** with lithium hydroxide [15]. In a manner similar to [24], firstly, a ‘cold’ Friedel–Crafts acylation in dichloromethane was attempted, in which oxalyl chloride was added at room temperature, and then reacted with aluminium trichloride at 0 °C. The resulting material was not soluble in cold dichloromethane/chloroform, but was found to be sufficiently soluble in hot chlorinated solvents. NMR spectroscopy in deuterated DMSO indicated that the ring closure proceeded on one side of the molecule. It was assumed that this species precipitated, preventing further reaction. We thus turned to a ‘hot’ Friedel–Crafts acylation, in which the reaction mixture was refluxed after the addition of oxalyl chloride, followed by removal of the volatiles under vacuum. Details of the synthesis are described in Supporting Information File 1. After fresh, anhydrous dichloromethane was added, the mixture was cooled to 0 °C in a water–ice bath, and resublimed aluminium trichloride was added. The mixture was allowed to warm to room temperature, and was then refluxed overnight [43]. The result was a dark violet/black material, which was not soluble in common organic solvents, thereby preventing NMR spectroscopy. Also, sublimation failed. However, the mass could be measured with MALDI mass spectrometry, and microanalysis results matched the theoretical values.

**Scheme 3:**
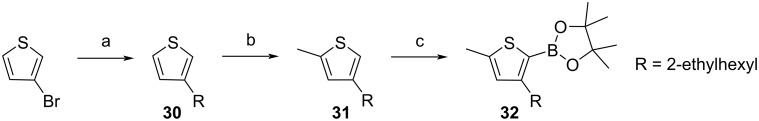
Synthesis of thiophene derivative **32**: a) Magnesium, 2-ethylhexylbromide, spatula tip iodine, anhydrous diethyl ether, 45 °C, 2 h, 3-bromothiophene, [1,3-bis(diphenylphosphino)propane]dichloronickel(II), 45 °C, 14.5 h, 40% [47]; b) *n*-butyllithium, 2,2,6,6-tetramethylpiperidine, anhydrous tetrahydrofuran, −80 °C, iodomethane, then rt, overnight, 86% [48]; c) *n*-butyllithium, anhydrous tetrahydrofuran, −5 °C, 1 h, then −78°C, trimethyl borate, then rt, overnight, 25% [49–50].

Since the ring-closure with a ‘hot’ Friedel–Crafts acylation [43] led to an insoluble material, we wanted to synthesise a soluble derivative by attaching 4-(2-ethylhexyl)-2-methylthiophene groups. This was done by reacting intermediate **27** with compound **32**, which was prepared as shown in Scheme 3.

In a manner similar to [49–50], 4-(2-ethylhexyl)-2-methylthiophene (**31**), which had been synthesised according to the published literature [47–48,51–52], was lithiated with *n*-butyllithium, and reacted with trimethyl borate [49–50]. Although a conversion could be detected with TLC, no boronic acid derivative of **31** could be isolated; it hydrolysed on the column back to the starting material. Thus, after reacting **31** with *n*-butyllithium and trimethyl borate, the obtained species was reacted *in situ* with pinacol to generate the corresponding boronic ester **32**, which could be isolated by column chromatography, in a manner similar to a reported procedure [49].

**Scheme 4:**
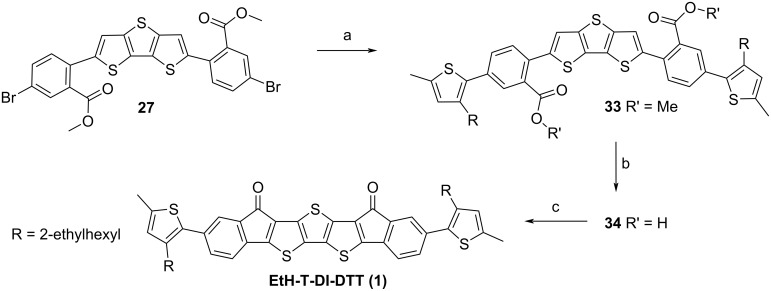
Synthesis of the soluble target structure **EtH-T-DI-DTT** (**1**): a) **32**, Pd(PPh_3_)_4_, K_2_CO_3_, THF, H_2_O, 70 °C, 45 h, 76% [40]; b) LiOH, THF, H_2_O, 70 °C [15], 6 d, 91%; c) oxalyl chloride, anhydrous DMF (cat.), anhydrous CH_2_Cl_2_, rt, 30 min, 40 °C, 4 h, AlCl_3_, anhydrous CH_2_Cl_2_, 0 °C, 15 min, rt, overnight, 60% [24,43].

In a manner similar to [40], intermediate **27** was reacted with **32** within a Suzuki–Miyaura coupling to achieve **33** (Scheme 4).

Intermediate **33** was then hydrolysed to the diacid **34** with lithium hydroxide [15]. Compound **34** was successfully reacted within a Friedel–Crafts acylation ring-closure reaction [24,43], in which the mixture was refluxed after oxalyl chloride addition, but cooled before the addition of AlCl_3_; the mixture was allowed to warm to room temperature and stirred overnight to complete the reaction. The obtained target substance **EtH-T-DI-DTT** (**1**) is readily soluble in a dichloromethane/petroleum ether mixture to enable column chromatography. A similar reaction sequence with an analogue of **33** without α-methyl groups at the terminal positions of the molecules was attempted; however, the corresponding ring closure failed. It is assumed that the reactive 5-position of thiophene underwent further reactions under the aggressive Friedel–Crafts acylation conditions [53].

### Physical properties

#### Absorption spectroscopy

**Figure 8:**
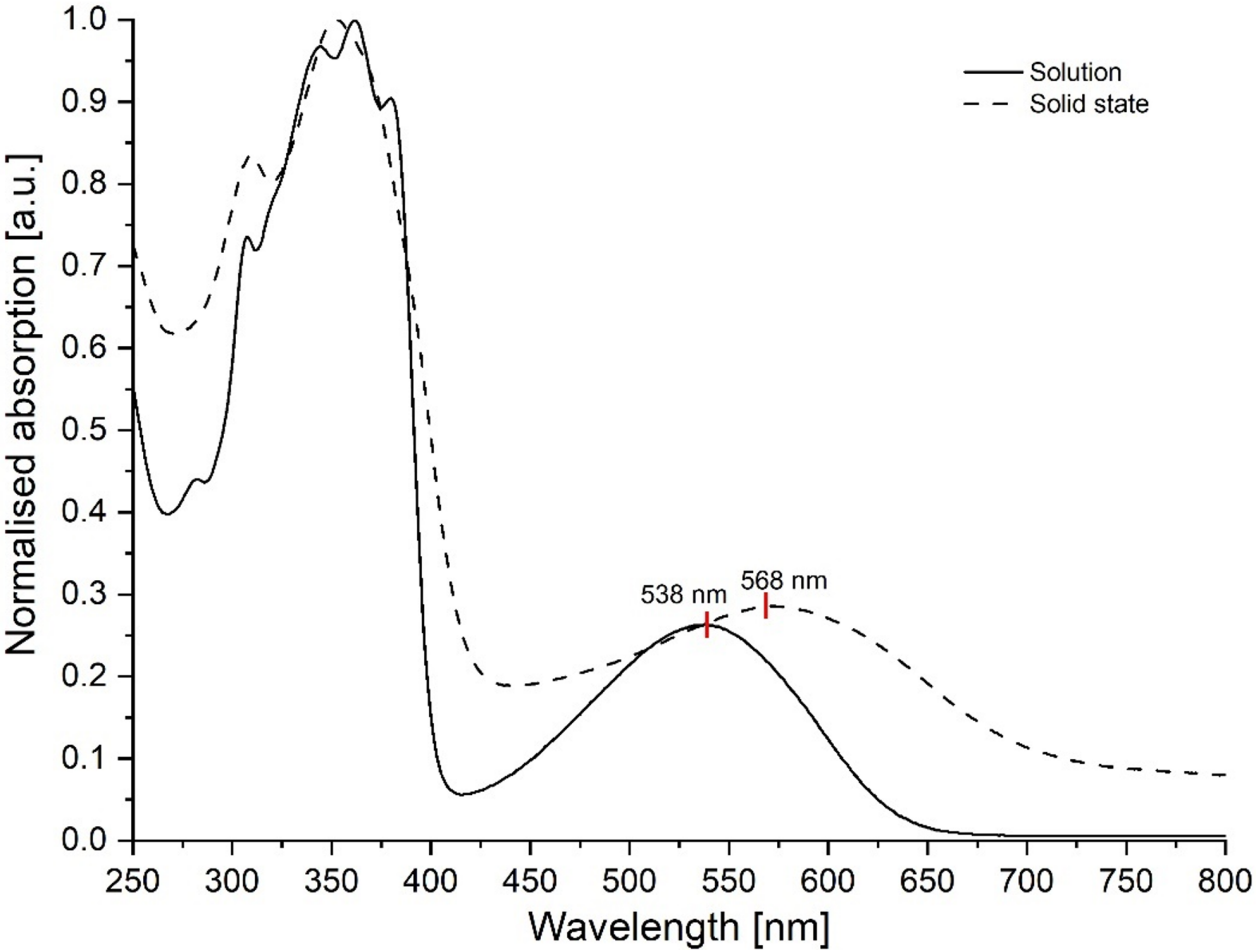
Normalised UV–vis spectra of **EtH-T-DI-DTT** in 10^−5^ M CH_2_Cl_2_ solution and in the solid state.

The target material **EtH-T-DI-DTT** (**1**) is a dark solid and forms dark violet solutions at low concentrations. Solution spectra of **EtH-T-DI-DTT** were measured in dichloromethane in 10^−5^ mol L^−1^ solutions. For solid-state measurements, **EtH-T-DI-DTT** was spin-coated from a chloroform solution on a quartz wafer. Both solution and solid-state spectra (Figure 8) show a main band at ca. 350 nm, which is due to a localised π–π* transition [54].

Interestingly, while the solution spectrum shows a clear fine structure, as expected for a rigid molecule [16], the solid-state spectrum does not; this could be due to different clusters of aggregates in the solid state. Also, both spectra show a broad and featureless lower energy band, which is considered to be an intramolecular charge transfer (ICT) transition [54]. Only the latter band is, in the solid state, shifted to lower energies compared to the one of the solution spectrum due to dipole–dipole interactions in the condensed phase, as seen for example in DPP derivatives [55], whose red-shifted ICT absorption band in the solid state, compared to solution state can be explained by quadrupole–quadrupole interactions.

From the onsets of the higher wavelength bands, the optical energy gap can be calculated by Equation 1 [56–57]:


[1] $$E_{g}=\frac{h\cdot c}{\lambda_{\text{on}}}$$

in which *h* is the Planck constant [58], *c* the speed of light [59], and λ_on_ the onset wavelength [60]. For the solution, λ_on_ was determined to be 638 nm, resulting in an optical energy gap of 1.94 eV. For the solid state, λ_on_ is shifted to higher wavelengths (701 nm), resulting in a smaller energy gap of *E*_g_ = 1.77 eV. The red shift is due to intermolecular interactions, which are known to lead to a narrowing of the energy gap [3,61]. With a concentration series, an extinction coefficient of ε_361 nm_ = 4.3 × 10^4^ L mol^−1^ cm^−1^ could be determined for the band at 361 nm, whilst for the band at 540 nm, a coefficient of ε_540 nm_ = 1.1 × 10^4^ L mol^−1^ cm^−1^ was determined [62].

#### Thermal properties

An uncorrected melting point of **EtH-T-DI-DTT** was measured to be 230 °C. The DSC curve, shown in Figure S19 in Supporting Information File 1, shows a maximum at 214 °C, and apart from that no other phase transitions, which means that the material does not change its phase below its melting point. The limits of thermal stability were recorded by a 5% mass loss at elevated temperatures, as determined by thermal gravimetric analysis (TGA), and found to be 406 °C, indicating a high thermal stability (Figure S18 in Supporting Information File 1).

#### Electrochemistry

**Figure 9:**
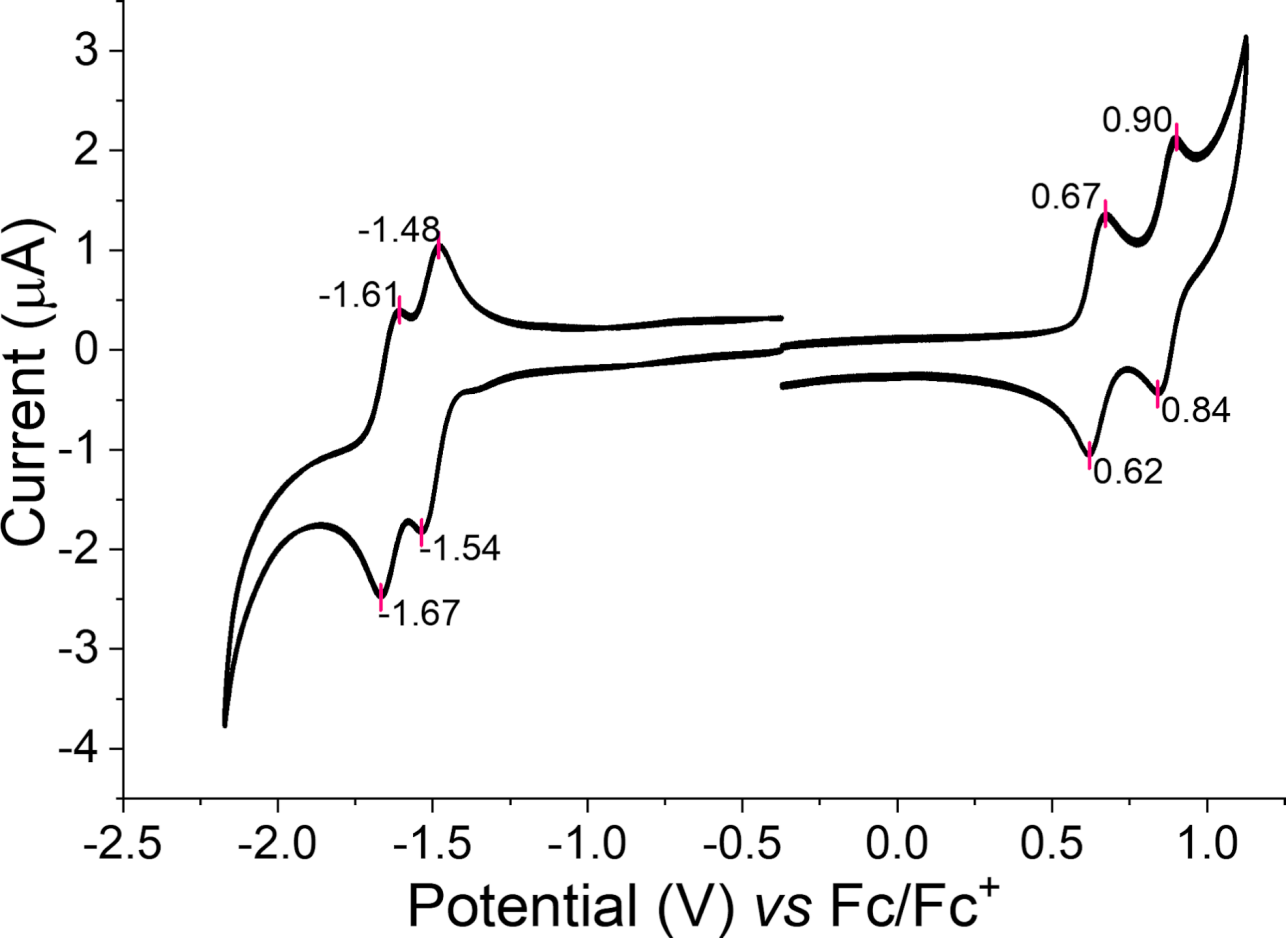
Cyclic voltammogram for **EtH-T-DI-DTT** (**1**), at a scan rate of 0.1 V s^−1^ using a Pt disk as the working electrode, Pt wire as the counter electrode, and Ag wire as the quasi-reference electrode in *ca*. 0.1 mM of the compound in CH_2_Cl_2_ with TBAPF_6_ (0.1 M) as the electrolyte [64].

Cyclic voltammetry (CV) was used to estimate the ionisation energy (IE) and the electron affinity (EA) of the title compound (Figure 9) [63].

CV was measured in a 10^−4^ M CH_2_Cl_2_ solution with 0.1 M tetra-*n*-butylammonium hexafluorophosphate (TBAPF_6_) added as electrolyte. A platinum disk electrode, a Pt wire, and an Ag wire were used as the working, counter and quasi-reference electrodes, respectively [64].

Regarding Koopman’s theorem, IE and EA correspond to the negative *E*_HOMO_ and *E*_LUMO_, respectively [63]. The frontier orbital energies can be calculated with the empirical formulae (Equation 2 and Equation 3) [65–66]:


[2] $$E_{\text{HOMO}}=-\left(E_{\text{ox}}-E_{{\text{Fc/Fc}}^{\text{+}}}+4.8\right)\left[\text{eV}\right]$$

[3] $$E_{\text{LUMO}}=-\left(E_{\text{red}}-E_{{\text{Fc/Fc}}^{\text{+}}}+4.8\right)\left[\text{eV}\right]$$

In which *E*_ox_ is the half-wave potential of the first oxidation signal, and *E*_red_ is the half-wave potential of the first reduction signal. Ferrocene was used as the internal standard and its HOMO was taken to be −4.8 eV [67]. With these values, the frontier orbital energy levels of **EtH-T-DI-DTT** could be estimated to be *E*_HOMO_ = −5.45 eV and *E*_LUMO_ = −3.29 eV, resulting in a single-particle gap [68] *E*_g_ of 2.2 eV. Compared to compound **3** published by our group [16], (*E*_HOMO_ = −5.4 eV, *E*_LUMO_ = −1.9 eV) the HOMO level of **EtH-T-DI-DTT** is similar, and the LUMO energy level is significantly more negative. This can be attributed to the presence of the electron-withdrawing keto groups [3,20].

In contrast to compounds **2**–**4**, which showed poor reversibility for oxidation and reduction, **EtH-T-DI-DTT** shows excellent electrochemical stability. Two sequential reversible oxidations can be seen in Figure 9, for the generation of a radical cation and dication at half-wave potential values of +0.65 V (Δ*E*_p_ = 0.05 V) and +0.87 V (Δ*E*_p_ = 0.06 V), respectively. Two reduction waves, corresponding to the radical anion and dianion, can be seen at the half-wave potentials of −1.51 V (Δ*E*_p_ = 0.06 V) and −1.64 V (Δ*E*_p_ = 0.06 V), respectively. UV–vis absorption spectroelectrochemistry (see Figures S20 and S21 in Supporting Information File 1) shows the evolution of the dication and dianion states with the longest wavelength absorption band extending into the near-IR with broad features, characteristic of highly delocalised bipolaron states [69].

#### Computation of structure

**Figure 10:**
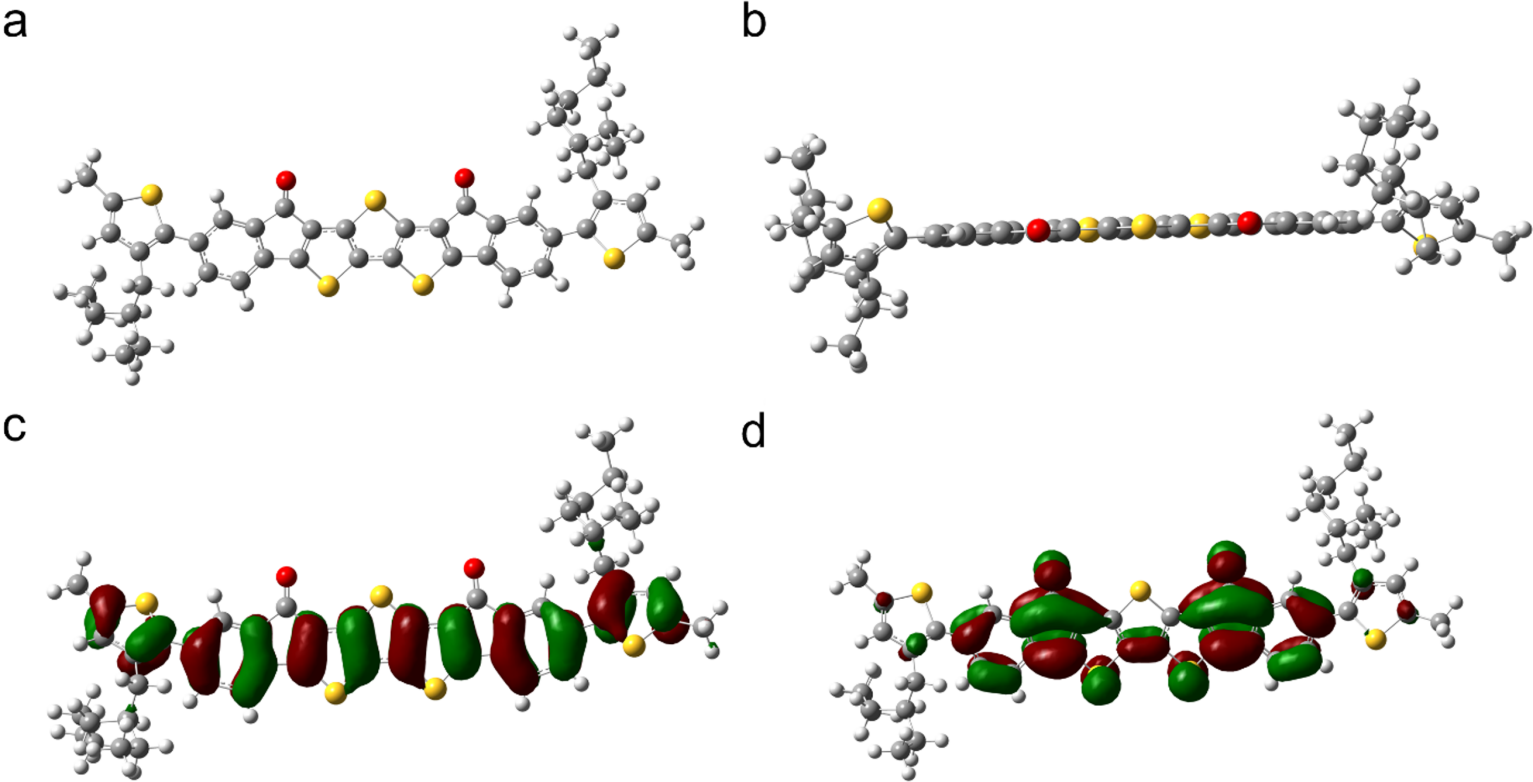
The structure of **EtH-T-DI-DTT** optimised on the B3LYP/6-311g(d,p) level of theory, viewed from the (a) top and (b) side-on, c) distribution of the HOMO in **EtH-T-DI-DTT**, d) distribution of the LUMO in **EtH-T-DI-DTT**.

Since all attempts to grow crystals of **EtH-T-DI-DTT** failed, we predicted the structure with a density functional theory (DFT) gas-phase optimisation using the B3LYP[70–71]/6-311g(d,p) [72] level of theory, using both the Gaussian09 [73] and Gaussview [74] programs. A frequency calculation showed that all frequencies are positive indicating that a minimum was found [75]. The calculations showed that the middle section consisting of seven fused ring systems is nearly planar (Figure 10).

The thiophene groups carrying the ethylhexyl chains are twisted out of plane, with a dihedral angle of *ca*. 40°.

Based on the optimised structure shown in Figure 10a and 10b, a subsequent cube calculation for the HOMO and LUMO was performed (Figures 10c and 10d, respectively).

Whilst the HOMO is distributed strongly in all ring systems, it spares all the sulfur atoms of the DTT core and the electron-withdrawing [20] keto groups. The LUMO is presented strongly in the seven fused rings, but is poorly represented in the outer ethylhexylthiophenes, which are electron-rich and twisted out of plane. The LUMO is strongly localised at the electron-deficient keto groups. Interestingly, neither the HOMO, nor the LUMO, is present at the central sulfur atom of the DTT motif.

#### Organic field-effect transistors

To estimate the charge carrier mobility from the saturation regime of the current–voltage plot, bottom gate/bottom contact (BG/BC) OFETs [76–77] were manufactured in a glovebox under inert conditions, using commercial wafers [78]. More details about device fabrication and applied wafers are described in Supporting Information File 1.

The devices were optimised by varying annealing temperatures [3,79–80], the concentration of the substrate, solvent choice [81], and application of self-assembled monolayers (SAM). SAMs are coated on the dielectric medium, improving surface roughness [82] and reduce interfacial defects [83].

Details about device fabrication are described in Supporting Information File 1. Device optimisation was necessary since only a weak field effect could be measured if no SAMs were used and no annealing was applied, with mobilities in the range 10^−7^–10^−9^ cm^2^ V^−1^ s^−1^. The best annealing temperature was 150 °C; mobilities deteriorated if higher or lower annealing temperatures were applied. The best hole mobility of a single device was measured upon annealing for 30 minutes at 150 °C with 1.33 × 10^−4^ cm^2^ V^−1^ s^−1^, using a solution of 10 mg mL^−1^ in CHCl_3_ and octadecyltrichlorosilane (OTS) as the SAM [82]. Averaged over seven devices on that wafer, an average hole mobility of 4.69 × 10^−5^ cm^2^ V^−1^ s^−1^ was measured. However, two devices were measured with mobilities in the 10^−6^ cm^2^ V^−1^ s^−1^ region on the wafer. The threshold voltage was determined to be −14.5 V ± 4.7 V, and an on/off ratio [76] of 10^2^–10^4^. The I_DS_–V_DS_ and I_DS_–V_GS_ plots of this device are shown in Figure S22 of Supporting Information File 1. No electron mobility was detectable.

In a similar study with chlorobenzene instead of chloroform, significantly worse mobilities were measured. Also, further increasing the concentration to 20 mg mL^−1^ CHCl_3_ led to lower mobilities.

## Conclusion

In summary, we have synthesised and characterised a novel diindenone-DTT compound, **EtH-T-DI-DTT**, consisting of seven, fused ring systems, with an electron-rich central DTT core, flanked by electron-withdrawing keto groups. Absorption studies in solution and in the solid state show strong aggregation of the molecules in films. **EtH-T-DI-DTT** shows excellent redox stability with two sequential reversible oxidations and two sequential reversible reduction waves. UV–vis spectroelectrochemistry reveals the absorption profiles of the dications and dianions as highly delocalised intermediate charged states. **EtH-T-DI-DTT** is readily soluble in organic solvents due to the ethylhexyl thiophene groups and has been applied in solution processed OFETs. A maximum hole mobility of 1.33 × 10^−4^ cm^2^ V^−1^ s^−1^ was measured for a single device. The isolation of the dibromide compound **29** provides the basis for the inclusion of this interesting molecule in larger conjugated structures or copolymers.

## Supporting Information

File 1 Synthetic details, and a detailed description of the analytical methods and device fabrication.
