# Supplementary material for: Introducing a new 7-ring fused diindenone-dithieno[3,2-b:2',3'-d]thiophene unit as a promising component for organic semiconductor materials
Source: Beilstein J Org Chem. 2022 Aug 1;18:944–55. doi: 10.3762/bjoc.18.94 (PMC9359197; doi:10.3762/bjoc.18.94)
Supplement: File 1 — Synthetic details, and a detailed description of the analytical methods and device fabrication. [file Beilstein_J_Org_Chem-18-944-s001.pdf]

BEILSTEIN JOURNAL OF ORGANIC CHEMISTRY

Supporting Information

for

Introducing a new 7-ring fused diindenone-dithieno[3,2-*b*:2',3'-*d*]thiophene unit as a promising component for organic semiconductor materials

Valentin H. K. Fell, Joseph Cameron, Alexander L. Kanibolotsky, Eman J. Hussien and Peter J. Skabara

Beilstein J. Org. Chem. **2022,** *18,* 944–955. doi:10.3762/bjoc.18.94

Synthetic details, and a detailed description of the analytical methods and device fabrication

License and Terms: This is a supporting information file under the terms of the Creative Commons Attribution License (https://creativecommons.org/licenses/by/4.0). Please note that the reuse, redistribution and reproduction in particular requires that the author(s) and source are credited and that individual graphics may be subject to special legal provisions.
The license is subject to the *Beilstein Journal of Organic Chemistry* terms and conditions: (https://www.beilstein-journals.org/bjoc/terms)

1. Methods

Solutions were degassed by freeze-degassing according to a published procedure [1]. If not otherwise stated, all chemicals from industrial vendors were used as received. *N*-Butyllithium was obtained from Sigma-Aldrich (2.5 M/hexane). Petroleum ether in the boiling point range 40–60 °C was used. For low temperature cooling below −20 °C, a mixture of liquid nitrogen and petroleum ether [2], for temperatures between 0 °C and −20 °C, a mixture of water–ice and sodium chloride was used [3]. Anhydrous tetrahydrofuran (THF), dichloromethane (CH_2Cl_2), diethyl ether, and toluene were obtained from a PureSolv solvent purification system (SPS) by innovative technology, Inc., using alumina as drying agent.

NMRs were referenced regarding their solvent peaks, according to published literature [4]. ^{1}H NMR spectra were measured at a frequency of 400 MHz, ^{13}C NMR at a frequency of 100 MHz, both with a Bruker-Spectrospin 400 Ultrashield™ 400. Elemental analyses were measured with an Exeter CE-440 Elemental Analyser. Electron ionisation (EI^+) mass spectrometry was performed with a JEOL MStation JMS-700, electrospray (ESI) spectrometry with a Bruker MicroTOFq. Also with the JEOL MStation JMS-700, FAB-MS was done using 3-nitrobenzyl alcohol as matrix.

MALDI-TOF spectrometry was performed with a Shimadzu Biotech Axima Confidence 2.9.3.20110624, reflection mode or a Shimadzu Biotech Axima CFR 2.8.3.20080616. All melting points are uncorrected and were measured with a Stuart Scientific Melting point apparatus. Absorption spectra were measured with a Shimadzu UV 2700, using quartz cuvettes with 1 cm path length for solutions. For solid-state measurements, films were obtained by spin-coating at 1000 rpm from chloroform solutions on a quartz wafer.

Electron affinities (EA) and ionisation energies (IE) were determined in CH_2Cl_2 solution by cyclic voltammetry using a CH Instruments 660E at a scan rate of 0.1 V s^{-1}, with an IR compensation before each run. Anhydrous dichloromethane was obtained from a SPS and was used to provide

the solutions with a substrate concentration of ca. 10^{-3} to 10^{-4} mol L^{-1}, using tetrabutylammonium hexafluorophosphate as electrolyte. The solutions were degassed with argon prior to the measurements. As a reference, the ferrocene redox couple Fc/Fc^+ was applied. As electrodes, a three-electrode setup was taken with platinum disk as working, platinum wire as counter, and silver wire as pseudo-reference electrodes [5]. As energy levels below vacuum for the ferrocene/ferrocenium (Fc/Fc^+) redox couple, 4.8 eV is taken [6].

Spectroelectrochemical measurements were performed by linking cyclic voltammetry (CH Instruments 660E Electrochemical workstation) and UV–vis (Shimadzu UV-2600 Spectrophotometer in the range 200–1200 nm) together. The experiment was performed using a solution-state spectroelectrochemistry kit, where the quartz cuvette path length is 1 mm. The electrodes used were platinum mesh, platinum wire, and silver wire as working electrode, counter electrode and reference electrode, respectively.

The electrodes were placed in the solution (0.1 mM monomer and 0.1 M electrolyte dissolved in dry DCM). In the oxidation process, the potential was initially increased gradually from 0 V to 1.5 V by measuring the absorption every 0.1 V. Then, the potential was decreased to zero gradually and absorption was measured. In the reduction process, the potential was decreased gradually from 0 V to −1.6 V then was increased gradually from −1.6 V to 0 V and the absorption was measured every 0.1 V.

Differential scanning calorimetry (DSC) measurements were performed with a Netzsch DSC 214 under nitrogen atmosphere. Thermogravimetric analysis (TGA) was measured with a Netzsch TG 209F3 under nitrogen atmosphere. Deionised water (DI-H_2O) was obtained from an ELGA Purelab option, Model OR007BPM1. Density Functional Theory (DFT) calculations were done with Gaussian09 [7], supported with Gaussview [8].

For OFETs, wafers, in which 230 $\pm$ 10 nm thick SiO_2 is the dielectric layer, and 30 nm Au + 10 nm indium tin oxide (ITO) is the adhesion layer as source/drain electrodes, and n-doped silicon is the gate, were purchased from Fraunhofer Institute for Photonic Microsystems, product No. 1301.

Each chip accommodates 16 transistors, each four with L = 2.5 μm, L = 5 μm, L = 10 μm, and L = 20 μm [9]. The width W is always 1 cm. Solutions of **EtH-T-DI-DTT** in chloroform and chlorobenzene were stirred overnight before coating. The chips were washed with deionised water, isopropanol, and acetone. If SAMs were used, octadecyltrichlorosilane (OTS) from toluene solution, or pentafluorobenzenethiol (PFBT) from ethanol solution, were drop-casted onto the chip. After drying with compressed air, the chip was brought into the glovebox. In the glovebox, the solution of **EtH-T-DI-DTT** was spin-coated with 1000 rpm onto the chip and, in some cases, annealed before testing.

2. Experimental

Dithieno[3,2-*b*:2’,3’-*d*]thiophene-2,6-diylboronic acid (25)

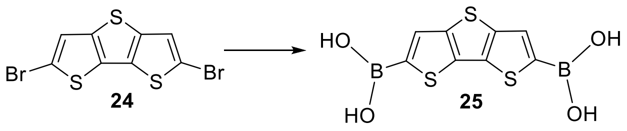

Two 500 mL three-necked flasks, one equipped with a stirring bar for the reaction (flask 1) and one for the SPS, were heated in the oven overnight and evacuated and refilled with argon three times. Flask 1 was charged with 4.02 g **24** (11.36 mmol, 1 equiv). The flask was evacuated and refilled with argon three times, and 200 mL anhydrous THF was injected, followed by one cycle of freeze-degassing. Over the course of 30 minutes, 10 mL *n*-butyllithium (2.5 M/hexane, 25 mmol, 2.2 equiv) was added at −90 °C, and the mixture was stirred at this temperature for another 20 minutes. The mixture was allowed to warm to −80 °C and stirred at this temperature for 20 minutes. The mixture was cooled to −90 °C and was stirred for 20 min at this temperature. Then, 16 mL triisopropylborate (69.3 mmol, 6.1 equiv) was injected rapidly in one portion. The mixture was allowed to warm to room temperature overnight, and 5 mL deionised water was injected. After stirring for 15 minutes, the mixture was poured onto 1 litre deionised water. Dropwise addition of 1 M hydrochloric acid until pH < 3 resulted in a grey precipitate to form. After extraction with diethyl ether (3 × 200 mL), the organic phases were combined. The solvent was evaporated, and the grey solid obtained was dried on a petri dish on air overnight, and dissolved in a small amount THF. After re-precipitation in petroleum ether, the obtained solid was filtered off and dried on air. Further drying in the desiccator led to 3.12 g (11.0 mmol, Y = 97%) of **25**, which was obtained as grey solid.

δ_H (DMSO-d$_6$, 400 MHz, ppm) 8.45 (4H, s), 7.95 (2H, s); δ_C ($CDCl_3$, 100 MHz, ppm) 143.6, 134.9, 129.4; m/z calc. for $C_8H_6B_2O_4S_3$: 283.96, found m/z (MALDI-TOF) 284.11; T_m: >360°C.

2,6-Bis(4,4,5,5-tetramethyl-1,3,2-dioxaborolan-2-yl)dithieno[3,2-*b*:2',3'-*d*]thiophene (26)

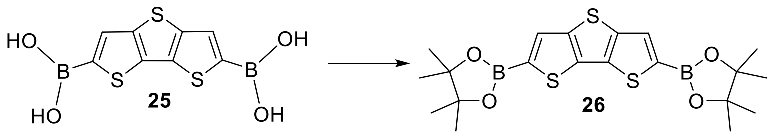

A condenser was attached to a 250 mL two-necked flask containing a stirring bar, and the apparatus was evacuated and refilled with argon once. The flask was charged with 4.17 g (14.7 mmol, 1 equiv) **25**, which had been dried in the desiccator overnight, and 3.86 g (32.7 mmol, 2.2 equiv) pinacol. After three cycles of evacuation and argon refilling, 140 mL SPS-toluene was added, followed by three cycles of freeze-degassing, and the solution was stirred at 115 °C for 21.5 h. The solvent was evaporated, and the residue was recrystallised from 200 mL hexane. After allowing to cool to rt, the solid was filtered and dried on air. After further drying in the desiccator, 5.21 g (11.6 mmol, Y = 79%) of **26** was isolated as grey solid.

δ_H ($CDCl_3$, 400 MHz, ppm) 7.76 (2H, s), 1.36 (24H, s), matching to published values [10]; δ_C ($CDCl_3$, 100 MHz, ppm) 144.9, 136.7, 130.4, 84.6, 24.9; m/z calc. for $C_{20}H_{26}B_2O_4S_3$+Na: 471.11, found m/z (ESI) 471.1072 $[M+Na]^+$; T_m = 286-288°C.

Dimethyl 6,6'-(dithieno[3,2-*b*:2',3'-*d*]thiophene-2,6-diyl)bis(3-bromobenzoate) (27)

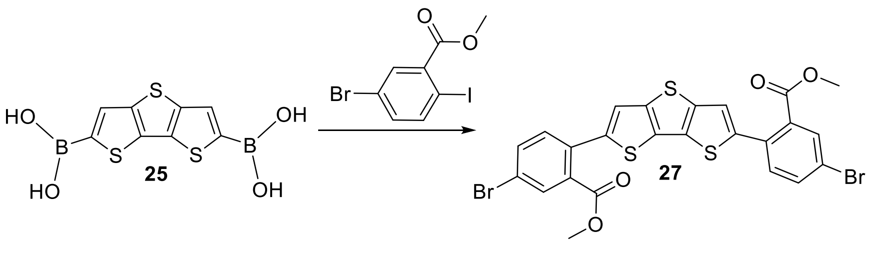

A 250 mL two-necked flask with a stirring bar and an attached condenser was evacuated and refilled with argon once. The flask was charged with 0.47 g (1.64 mmol, 1 equiv) **25** and 2.14 g

(6.27 mmol, 3.8 equiv) methyl 5-bromo-2-iodobenzoate, and again evacuated and refilled with argon three times. Then, 80 mL SPS-THF and 0.70 g Aliquat 336® were added, followed by one cycle freeze-degassing. Tetrakis(triphenylphosphine)palladium(0) (0.20 g, 0.18 mmol, 0.11 equiv) was added, followed by two more cycles of freeze-degassing. Then, 10 mL of a 3 × freeze-degassed potassium carbonate solution (0.91 g K_2CO_3 in 6.57 mmol, 4 equiv) was added to the mixture, which was then stirred at 70 °C for 40 h. The solvents were removed on the rotary evaporator, and the residue was purified by column chromatography twice (eluent: petroleum ether/dichloromethane/triethylamine 5:4.9:0.1). The solid was dried on air and in the desiccator. The product was obtained as yellow solid (0.28 g, 0.46 mmol, Y = 28%).

δ_H ($CDCl_3$, 400 MHz, ppm) 7.92 (2H, s), 7.67 (2H, d, *J* = 8.24 Hz), 7.42 (2H, d, *J* = 8.42 Hz), 7.25 (2H, s), 3.79 (6H, s); δ_C ($CDCl_3$, 100 MHz, ppm) 167.5, 141.6, 141.3, 134.4, 133.2, 133.1, 132.9, 132.8, 131.5, 122.4, 120.3, 52.9; m/z calc. for $C_{24}H_{14}Br_2O_4S_3$: 621.84, found m/z (EI^+) 621.8401; $[M]^+$; Elemental analysis calc. for $C_{24}H_{14}Br_2O_4S_3$: C, 46.32; H, 2.27%, found: C, 46.25; H, 2.13% and C, 46.25; H, 2.19%, in average $\bar{C}$, 46.25; $\bar{H}$, 2.16%; T_m = 158-160°C.

6,6’-(Dithieno[3,2-*b*:2’,3’-*d*]thiophene-2,6-diyl)bis(3-bromobenzoic acid) (28)

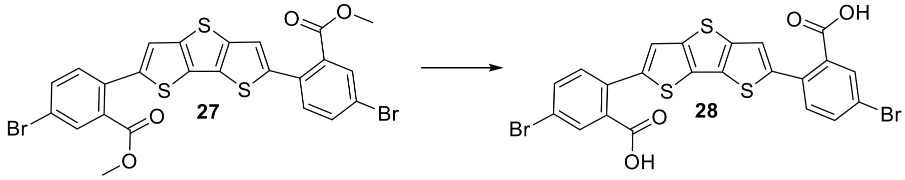

A 250 mL flask with a stirring bar and an attached condenser was charged with 0.53 g (0.85 mmol, 1 equiv) **27**, and was evacuated and refilled with argon three times. SPS-THF (20 mL) was injected, followed by one cycle freeze-degassing. Then, a lithium hydroxide solution (0.13 LiOH, 5.33 mmol, 6.3 equiv in 35 mL deionised water), which had been freeze-degassed once, was

injected into the flask. The mixture was heated to 70 °C, and stirred at this temperature for 24.5 hours, then allowed to cool to room temperature. The THF was evaporated, and 10 mL 1 M hydrochloric acid was added, leading to a yellow precipitate, which was filtered off and washed with 200 mL deionised water, 100 mL chloroform, and dried on air. After further drying in the desiccator, 0.50 g (0.83 mmol, Y = 98%) of **28** were obtained as yellow solid.

δ_H (DMSO-d_6, 400 MHz, ppm) 7.88 (2H, d, J = 1.78), 7.80 (2H, dd, J_1 = 8.26 Hz, J_2 = 1.78 Hz), 7.61 (2H, s), 7.54 (2H, d, J = 8.27 Hz); δ_C (DMSO-d_6, 100 MHz, ppm) 168.0, 141.2, 141.2, 135.0, 133.7, 132.7, 131.6, 131.4, 130.3, 121.5, 121.1; m/z calc. for $C_{22}H_{10}Br_2O_4S_3$: 593.81, found m/z (MALDI-TOF): 593.52 $[M]^+$; T_m = 310°C (assumed decomposition).

Diindenone-DTT (29)

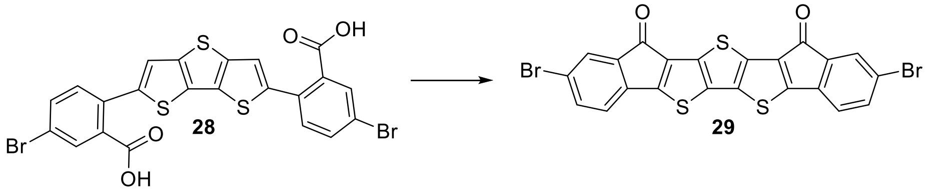

Intermediate **28** was dried in the desiccator overnight, aluminium trichloride was resublimed at 180 °C. A 250 mL three-necked flask with a stirring bar (flask 1), a condenser, a cooling trap, and a 250 mL two-necked flask (for SPS) were heated in the oven overnight. The three-necked flask was connected with the condenser, which was connected to a Schlenk-line *via* the three-way-tap 1, which other opening was connected to an additional oil bubbler. One of the outer necks of the three-necked flask was connected with the cooling trap *via* the three-way-tap 2. The cooling trap was also connected to the Schlenk line. The following picture shows the apparatus:

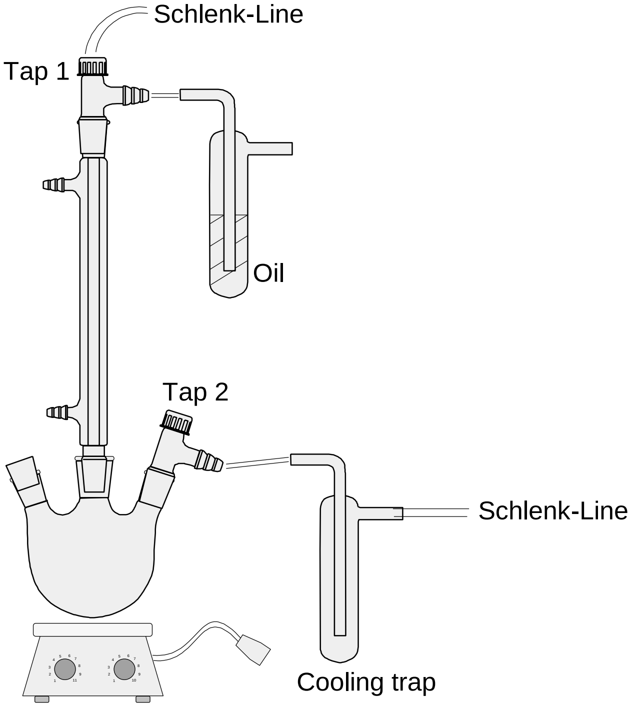

Figure S1: Apparatus used for the ‘hot’ Friedel–Crafts acylation.

After the apparatus was evacuated and refilled with argon three times, flask 1 was charged with 0.49 g (0.82 mmol, 1 equiv) **28**. The apparatus was again evacuated and refilled with argon (Tap 1 closed, Tap 2 open) three more times. Then, 50 mL anhydrous, 1 × freeze-degassed dichloromethane and 5 drops anhydrous *N*,*N*-dimethylformamide were injected. Afterwards, 0.3 mL oxalyl chloride (3.55 mmol, 4.3 equiv) were injected (Tap 1 open, Tap 2 closed) to the yellow, turbid mixture. The solution was stirred for 30 minutes until it was clear. The solution was stirred at 50 °C for three hours, and allowed to cool to room temperature (Tap 1 open, Tap 2 closed). The volatiles were evaporated (Tap 1 closed, Tap 2 open), and were trapped in the cooling trap filled with liquid nitrogen.

After that, the solid was dried under vacuum for 1.5 h at 35 °C. After 120 mL anhydrous, 1 × freeze-degassed dichloromethane was added, the red solution was cooled to 0 °C in an ice–water bath. Then, 1.26 g (9.46 mmol, 11.6 equiv) aluminium trichloride was added in one portion. There was a rapid change to dark colour, and the mixture was stirred for 15 minutes at

0 °C, and another 15 min without ice bath, then heated to 40 °C, and stirred at this temperature for 13 h. The dichloromethane was evaporated. Addition of 300 mL of a 1 M hydrochloric in ice–water solution led to the formation of a dark solid, which was filtered off. The solid was washed with 400 mL deionised water, 200 mL methanol, and 100 mL chloroform. The solid was then boiled in 150 mL chloroform, and filtered-off whilst still being hot. The insoluble solid was dried on air. Further drying in the desiccator led to 0.33 g (0.58 mmol, Y = 72%) of **29**, which was obtained as dark purple/black powder.

m/z calc. for $C_{22}H_6Br_2O_2S_3$: 555.79, found m/z (MALDI-TOF) 557.17 $[M]^+$; Elemental analysis calc. for $C_{22}H_6Br_2O_2S_3$: C, 47.33; H, 1.08%, found: C, 47.32; H, 1.31% and C, 47.49; H, 1.29%, in average $\overline{C}$, 47.41; $\overline{H}$, 1.30%; No melting observable below 360°C.

3-(2-Ethylhexyl)-5-methyl-5-(4,4,5,5-tetramethyl-1,3,2-dioxaborolan-2-yl)-thiophene (32)

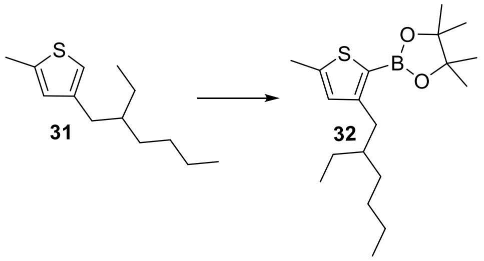

A 250 mL two-necked flask with a stirring bar and one 250 mL two-necked flask for solvent were heated in the oven overnight, and were evacuated and refilled with argon three times. The flask with the stirring bar was charged with 2.17 g (10.30 mmol, 1 equiv) 4-(2-ethylhexyl)-2-methylthiophene (**31**). After 50 mL anhydrous THF were injected, the solution was freeze-degassed once. In a NaCl/water/ice bath, the flask was cooled to −5 °C. Over the course of 15 min, 5.5 mL *n*-butyllithium (13.75 mmol, 1.3 equiv) was injected. The mixture was stirred at

−5 °C for one hour, and was cooled to −78°C, followed by a rapid injection of 4 mL trimethyl borate (ca. 35.88 mmol, 3.5 equiv). The mixture was allowed to warm to room temperature overnight. The septum was removed, and 6.31 g pinacol (53.36 mmol, 5.2 equiv) were added in one portion, and the flask was closed again. Since the viscosity increased strongly after a few hours, 50 mL THF were added. The mixture was stirred overnight, and was poured into 500 mL deionised water. Extraction was done with diethyl ether (3 × 100 mL). The residue was purified by column chromatography (eluent: petroleum ether). The product was obtained as colourless oil (0.86 g, 2.56 mmol, Y = 25%).

δ_H ($CDCl_3$, 400 MHz, ppm) 6.65 (1H, s), 2.72 (2H, d, *J* = 7.16 Hz), 2.47 (3H, s), 1.53 (1H, septet, *J* = 6.17 Hz), 1.31 (12H, s), 1.29-1.20 (8H, m), 0.91-0.83 (6H, m); δ_C ($CDCl_3$, 100 MHz, ppm) 154.4, 145.9, 130.0, 83.5, 41.5, 34.6, 32.7, 29.0, 25.7, 24.9, 24.9, 23.3, 15.6, 14.3, 10.9; m/z calc. for $C_{19}H_{33}BO_2S$+Na: 359.22, found m/z (ESI) 359.2187 $[M+Na]^+$; Elemental analysis calc. for $C_{19}H_{33}BO_2S$: C, 67.85; H, 9.89%, found: C, 67.75; H, 9.91% and C, 67.85; H, 9.89%, in average $\bar{C}$, 67.80; $\bar{H}$, 9.90%.

Intermediate 33

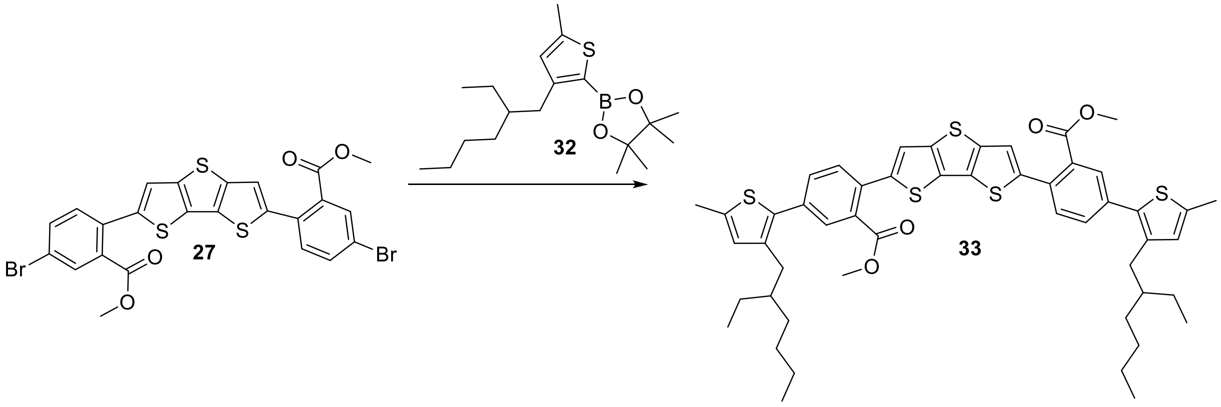

A 250 mL two-necked flask with a stirring bar was attached to a condenser. After addition of 0.55 g of **27** (0.89 mmol, 1 equiv), the flask was evacuated and refilled with argon three times. Then, compound **32** (1.16 g, 3.43 mmol, 3.9 equiv) and 90 mL SPS-THF were added, followed by one cycle of freeze-degassing. Afterwards, 0.20 g tetrakis(triphenylphosphine)palladium(0) (0.18 mmol, 0.20 equiv) was added, followed by two more cycles freeze-degassing. After injection of 10 mL of a 3 × freeze-degassed, aqueous K_2CO_3 solution (1.29 g, 9.36 mmol, 10.6 equiv), the mixture was heated to 70 °C and stirred at this temperature for 45 h. After cooling to room temperature, the aqueous phase was removed with a separation funnel. The organic solvent was evaporated, and the residue was purified by column chromatography six times (petroleum ether/dichloromethane 1:1). Removal of solvent and drying on a petri dish led to 0.60 g (0.68 mmol, Y = 76%) of the product, a yellow resin.

δ_H ($CDCl_3$, 400 MHz, ppm) 7.82 (2H, s), 7.56 (4H, s), 7.29 (2H, s), 6.65 (2H, s), 3.81 (6H, s), 2.58 (4H, d, *J* = 7.07 Hz), 2.50 (6H, s), 1.58 (2.4H[a], septet, *J* = 5.56 Hz), 1.35-1.12 (17.2H[b], m), 0.88-0.78 (12H, m); δ_C ($CDCl_3$, 100 MHz, ppm) 168.8, 142.6, 141.2, 138.9, 138.8, 135.6, 134.2, 132.4, 131.9, 131.7, 131.4, 130.5, 128.8, 120.0, 52.7, 40.7, 33.0, 32.7, 28.8, 25.9, 24.0, 23.2, 15.5, 14.3, 11.0; m/z calc. for $C_{50}H_{56}O_4S_5$+Na: 903.27, found m/z (ESI) 903.2674 $[M+Na]^+$; Elemental

analysis calc. for $C_{50}H_{56}O_4S_5$: C, 68.14; H, 6.41%, found: C, 67.95; H, 6.35% and C, 67.99; H, 6.40%, in average $\bar{C}$, 67.97; $\bar{H}$, 6.38%.

[a]: Integral overlaps with H_2O-signal.[4]

[b]: Integral overlaps with pet. ether and/or ^{1}H-grease signal.[4]

Intermediate 34

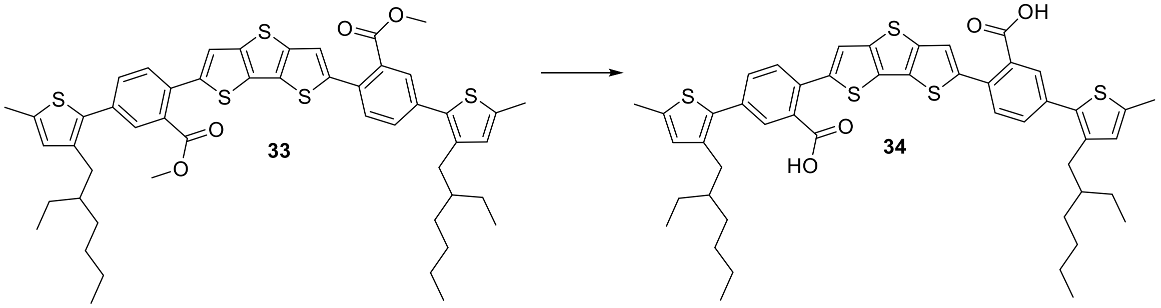

A 100 mL two-necked flask with an attached condenser was evacuated and refilled with argon once. The flask was then filled with a solution of **33** in 25 mL SPS-THF (0.54 g, 0.61 mmol, 1 equiv). After one cycle of freeze-degassing, a lithium hydroxide monohydrate solution (1.69 g, 40.3 mmol, 66 equiv, in 25 mL deionised water) was injected, which had been freeze-degassed once. The mixture was heated to 70 °C and stirred for six days. The tetrahydrofuran was evaporated, and the residue was poured into 200 mL 2 M hydrochloric acid solution, leading to the formation of a yellow solid. Since **33** decomposes under acidic conditions, the mixture was stirred only for 5 minutes, and then filtered off, and washed with 300 mL deionised water. The residue, a sticky, brown slime, was dissolved in dichloromethane and re-precipitated in petroleum ether on a petridish. The petroleum ether was allowed to dry (it was not filtered off), and the yellow, solid residue was dried on air and in the desiccator, resulting in 0.48 g of **34** (0.58 mmol, Y = 91%).

δ_H ($CDCl_3$, 400 MHz, ppm) 8.05 (2H, s), 7.56, (4H, dd, J_1 = 23.74 Hz, J_2 = 7.74 Hz), 7.28 (2H, s), 6.64 (2H, s), 2.57 (4H, d, J = 6.34 Hz), 2.49 (6H, s), 1.57 (2.70H[a], broad s), 1.33-1.13 (18H[b], m), 0.89-0.76 (m, 12.9[b]H); δ_C ($CDCl_3$, 100 MHz, ppm) 173.9, 142.0, 141.4, 139.0, 138.9, 135.6, 134.0, 133.6, 132.9, 132.3, 132.0, 131.4, 129.9, 128.8, 120.7, 40.7, 33.0, 32.7, 28.8, 25.9, 23.2, 15.5, 14.3, 11.0; m/z calc. for $C_{48}H_{52}O_4S_5$: 852.25, found m/z (FAB+) 852.2219 $[M]^+$; Elemental analysis calc. for $C_{48}H_{52}O_4S_5$: C, 67.57; H, 6.14%, found: C, 67.65; H, 6.25% and C, 67.58; H, 6.15%, in average $\bar{C}$, 67.62; $\bar{H}$, 6.20%; T_m = 170-172°C.

[a] Integral overlaps with water-signal.[4]

[b]: Integral overlaps with pet. ether and/or ^{1}H-grease signal.[4]

EtH-T-DI-DTT (1)

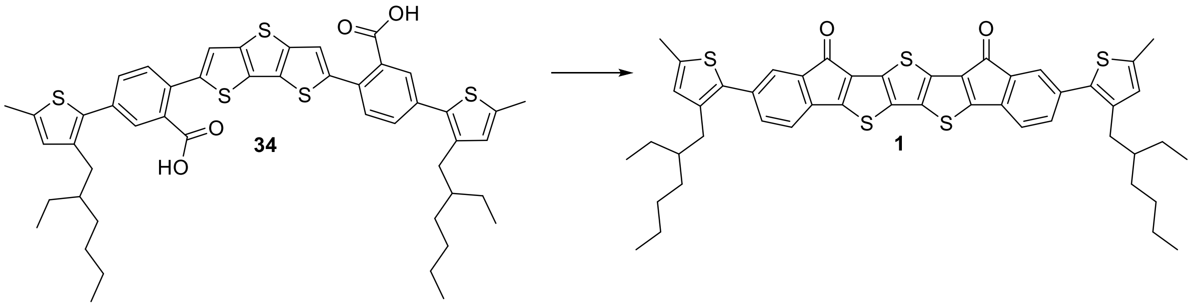

Aluminium trichloride was resublimed at 250 °C and was stored in a dried, Ar-filled glass ampoule. Diacid **34** was dried in the desiccator overnight.

A 250 mL three-necked flask with stirring bar, a condenser, a cooling trap, and a 250 mL two-necked flask for solvent were heated in the oven overnight, and attached according to Figure S1. After three cycles of evacuation and Ar-refill, the 250 mL three-necked flask with stirring bar was charged with 0.46 g (0.53 mmol, 1 equiv) **34**, and was evacuated and 3 × refilled with argon (tap 1 open, tap 2 closed). Afterwards, 40 mL anhydrous, 1 × freeze-degassed CH_2Cl_2 and 5 drops

anhydrous DMF were added. Keeping tap 1 open and tap 2 closed, 0.2 mL oxalyl chloride (2.36 mmol, 4.4 equiv) was added dropwise into the clear, yellow solution, leading to an exothermic reaction. The mixture was stirred at rt for 30 minutes, and was then refluxed at 40 °C for 4 hours. The mixture was allowed to cool to rt, and the volatile substances were removed under reduced pressure (Tap 1 closed, Tap 2 open). The remaining orange solid was dried under vacuum for 1.5 h at 40 °C. Then, 100 mL anhydrous 1 × freeze-degassed CH_2Cl_2 was added, and the mixture was cooled to 0 °C. At 0 °C, 1.70 g (12.7 mmol, 23.8 equiv) $AlCl_3$ was added in one portion, leading to a quick change to dark colour. The mixture was stirred overnight at rt. The solvent was removed with a rotary evaporator, 300 mL 1 M ice–hydrochloric acid was added, and the mixture was stirred for 20 min, followed by extraction with dichloromethane (3 × 150 mL). The dichloromethane phases were washed with 1 × 200 mL 5% aqueous $NaHCO_3$ solution, and the CH_2Cl_2 was evaporated. The crude product was purified by column chromatography, using the eluent mixture petroleum ether/dichloromethane 4:6. The solvent was evaporated, and the product was boiled in methanol, and was filtered off whilst still being hot. Drying in the desiccator overnight led to (0.26 g, 0.32 mmol, Y = 60%) **EtH-T-DI-DTT**, obtained as dark violet powder.

δ_H ($CDCl_3$, 400 MHz, ppm) 7.51 (2H, d, *J* = 1.39 Hz), 7.35 (2H, dd, J_1 = 7.66 Hz, J_2 = 1.64 Hz), 7.12 (2H, d, *J* = 7.61 Hz), 6.61 (2H, d, *J* = 0.79 Hz), 2.53 (4H, d, *J* = 7.15 Hz), 2.47 (6H, s), 1.55 (2H, p, *J* = 5.75 Hz), 1.28-1.12 (16H, m), 0.83 (6H, t, *J* = 7.08 Hz), 0.78 (6H, t, *J* = 7.60 Hz);

δ_C ($CDCl_3$, 100 MHz, ppm) 185.0, 160.3, 138.9, 138.5, 137.2, 136.6, 136.1, 135.5, 134.7, 134.3, 134.0, 133.7, 128.9, 124.7, 119.4, 40.7, 33.2, 32.7, 28.8, 25.9, 23.2, 15.5, 14.3, 10.9; m/z calc. for $C_{48}H_{48}O_2S_5$: 816.23, found m/z (FAB+) 816.1576 $[M]^+$; Elemental analysis calc. for $C_{48}H_{48}O_2S_5$: C, 70.55; H, 5.92%, found: C, 70.69; H, 5.99% and C, 70.85; H, 5.85%, in average $\bar{C}$, 70.77; $\bar{H}$, 5.92%; T_m = 230°C.

Tetrakis(**triphenylphosphine**)**palladium**(**0**)[11]

Three 250 mL two-necked flasks, one with stirring bar and one for solvent, a 100 mL one-necked flask and a filter-column were heated in the oven overnight. The 250 mL flask with stirring bar and one 250 mL flask without stirring bar were connected to the filter-column, and the glassware was evacuated and refilled with argon three times. The 250 mL flask with stirring bar was charged with 1.02 g palladium dichloride (5.76 mmol, 1 equiv) and 7.55 g triphenylphosphine (28.8 mmol, 5.0 equiv). The flask was again evacuated and refilled with argon three times, and 80 mL anhydrous dimethylsulfoxide was injected. After one cycle of freeze-degassing, the mixture was heated to 160 °C for 30 minutes, the mixture turned into a clear, red solution in that time. The heating pan was removed, followed by a rapid injection of 1.2 mL hydrazine monohydrate (24.6 mmol, 4.3 equiv). The mixture was stirred until it reached rt, allowing a yellow precipitate to form. The DMSO was removed *via* the filter-column. The yellow powder was firstly washed with anhydrous methanol (3 × 30 mL) and then with anhydrous diethyl ether (3 × 30 mL). The product was transferred into the 100 mL one-necked flask, and dried under vacuum for 90 minutes. The title catalyst was used as received without further purification or characterisation (6.30 g, 5.45 mmol, Y = 95%).

3. NMR spectroscopy

Intermediate 25

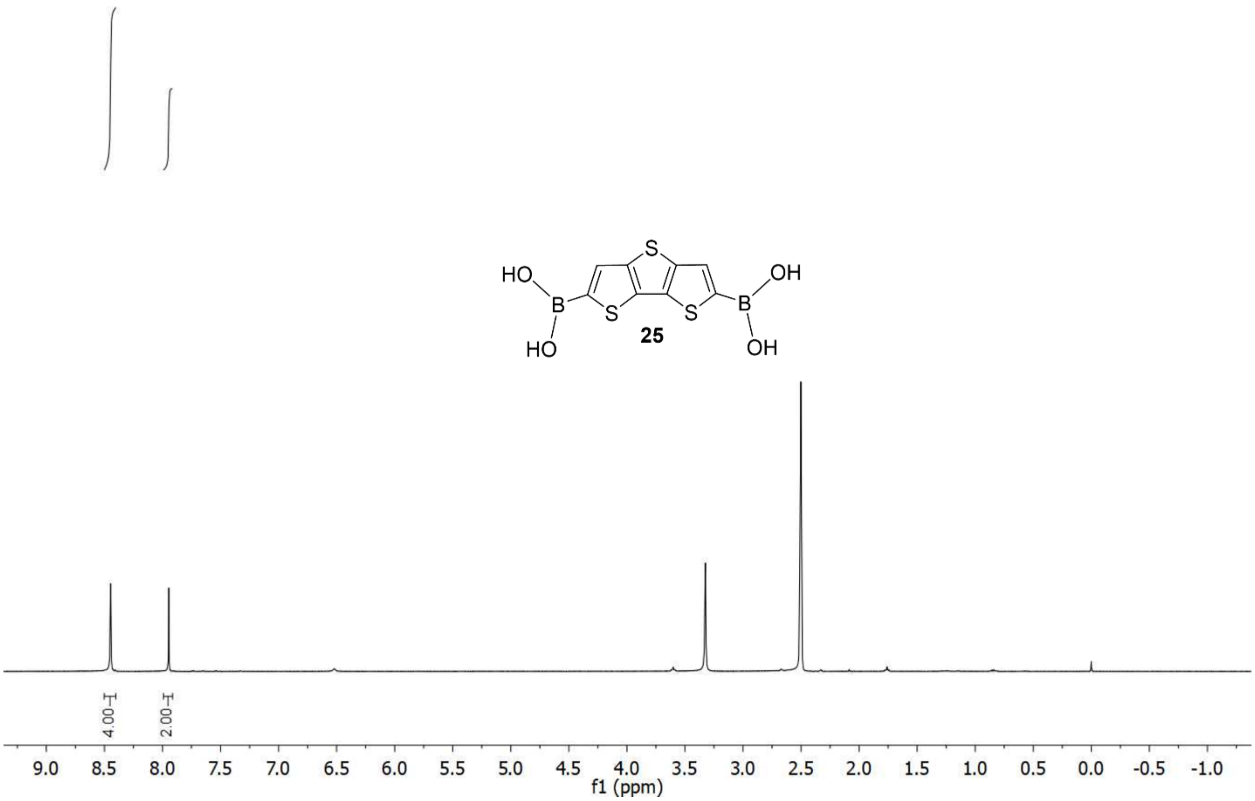

Figure S2: ^{1}H NMR spectrum of **25** in DMSO-d_6.

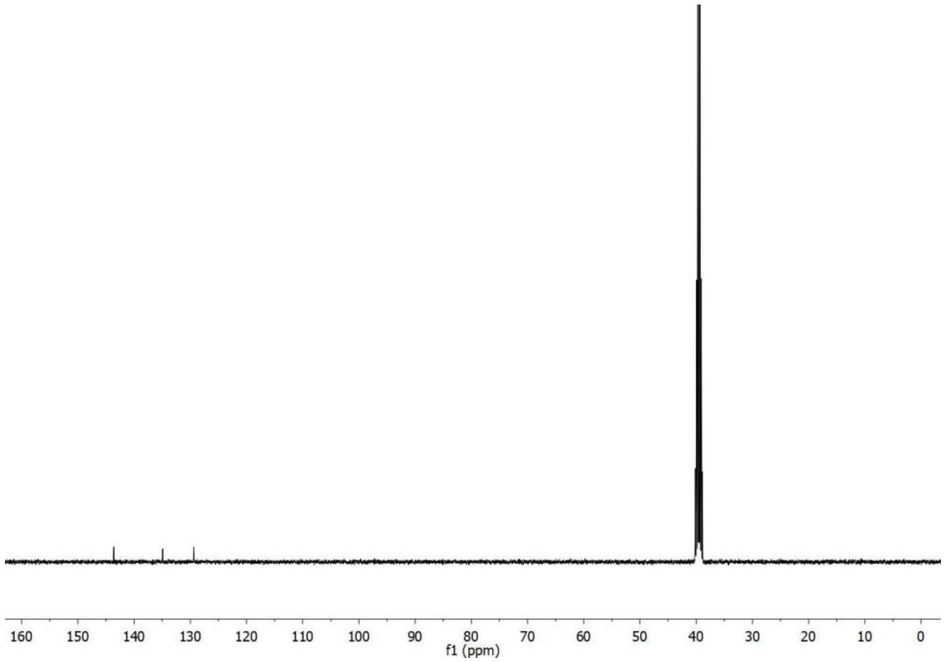

Figure S3: ^{13}C NMR spectrum of **25** in DMSO-d_6.

Intermediate 26

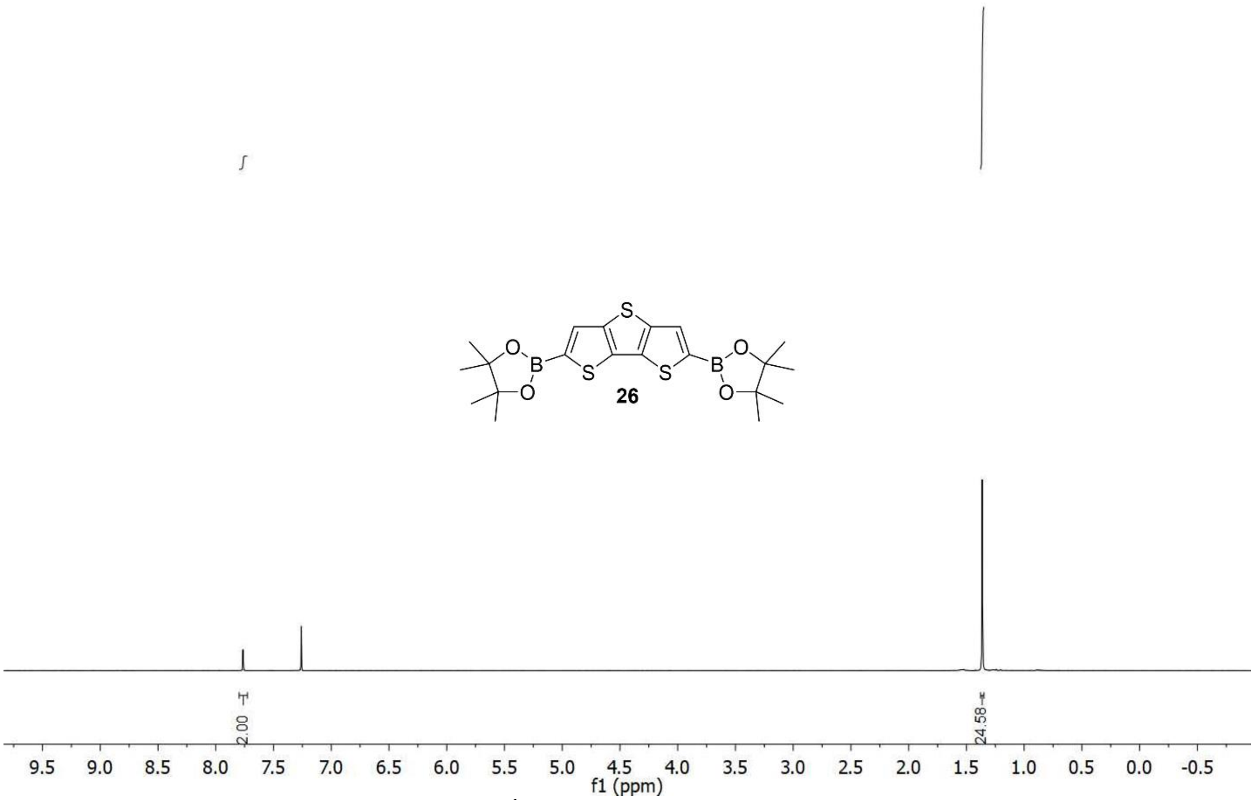

Figure S4: ^{1}H NMR spectrum of **26** in $CDCl_3$.

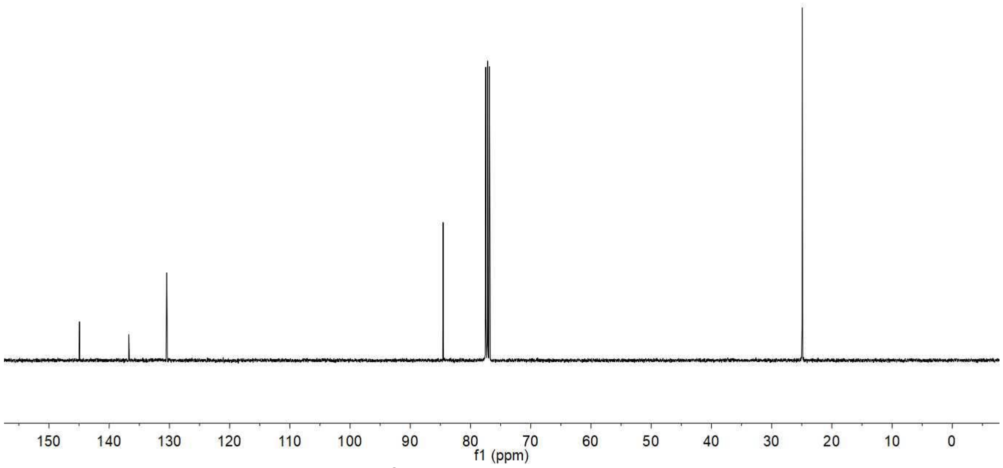

Figure S5: ^{13}C NMR spectrum of **26** in $CDCl_3$.

Intermediate 27

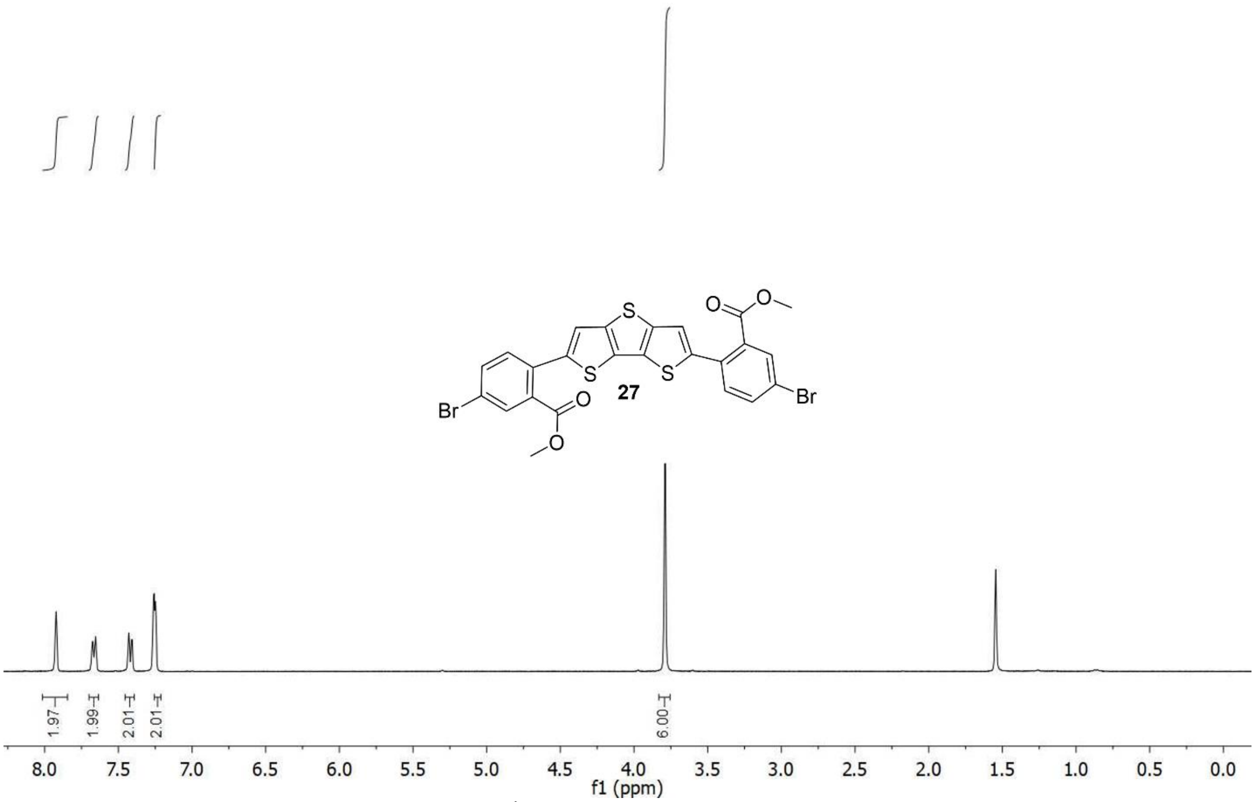

Figure S6: ^{1}H NMR spectrum of **27** in $CDCl_3$.

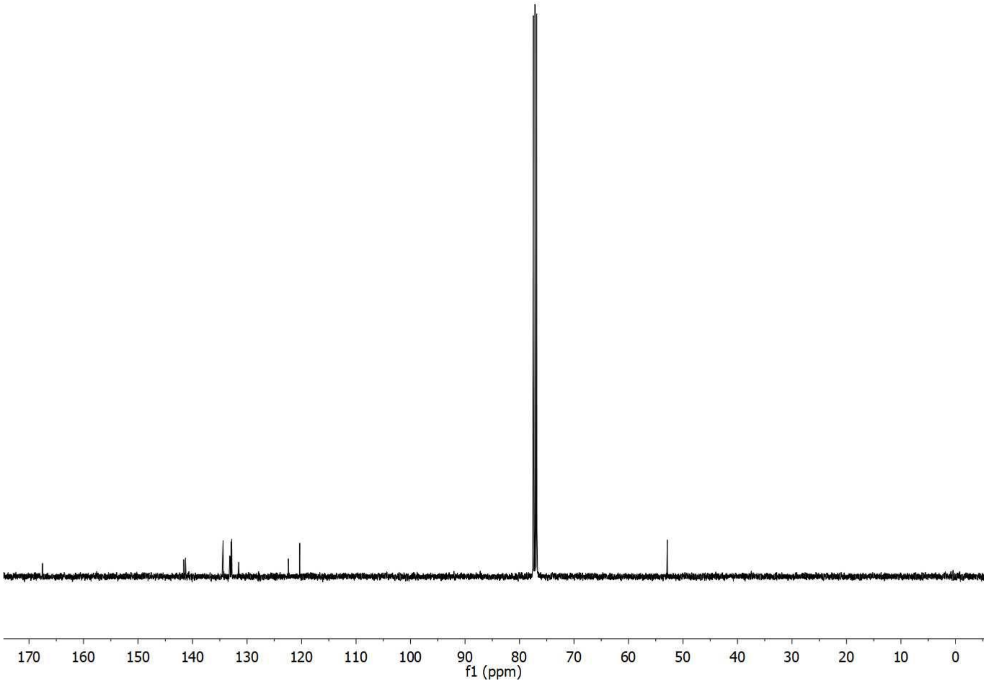

Figure S7: ^{13}C NMR spectrum of **27** in $CDCl_3$.

Intermediate 28

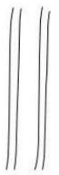

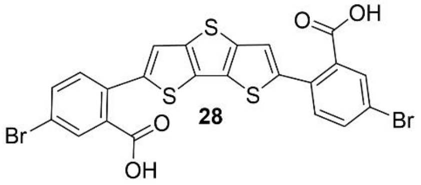

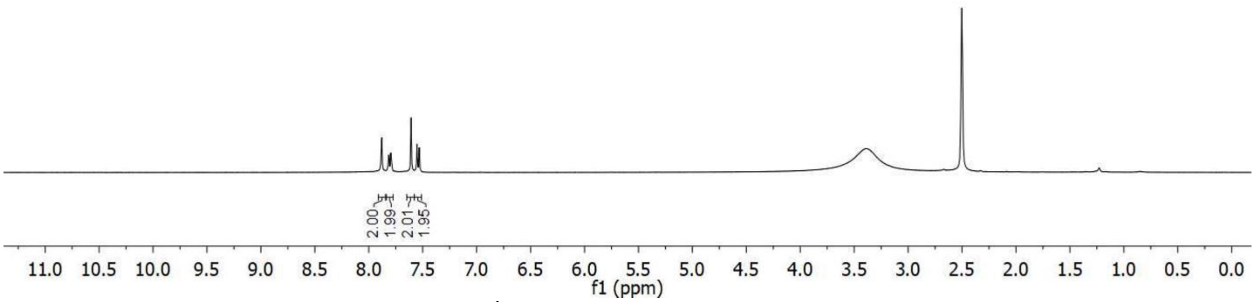

Figure S8: ^{1}H NMR spectrum of **28** in DMSO-d_6.

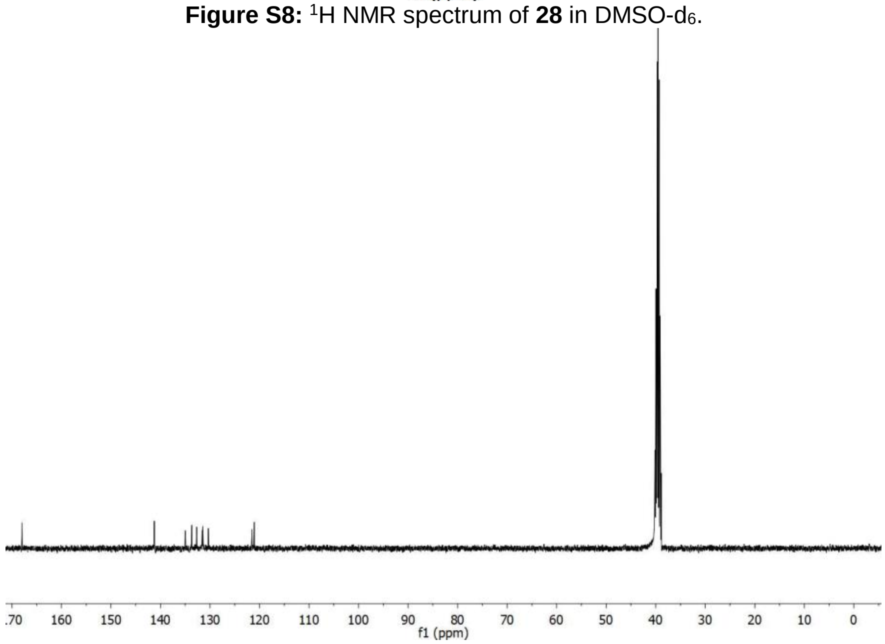

Figure S9: ^{13}C NMR spectrum of **28** in DMSO-d_6.

Intermediate 32

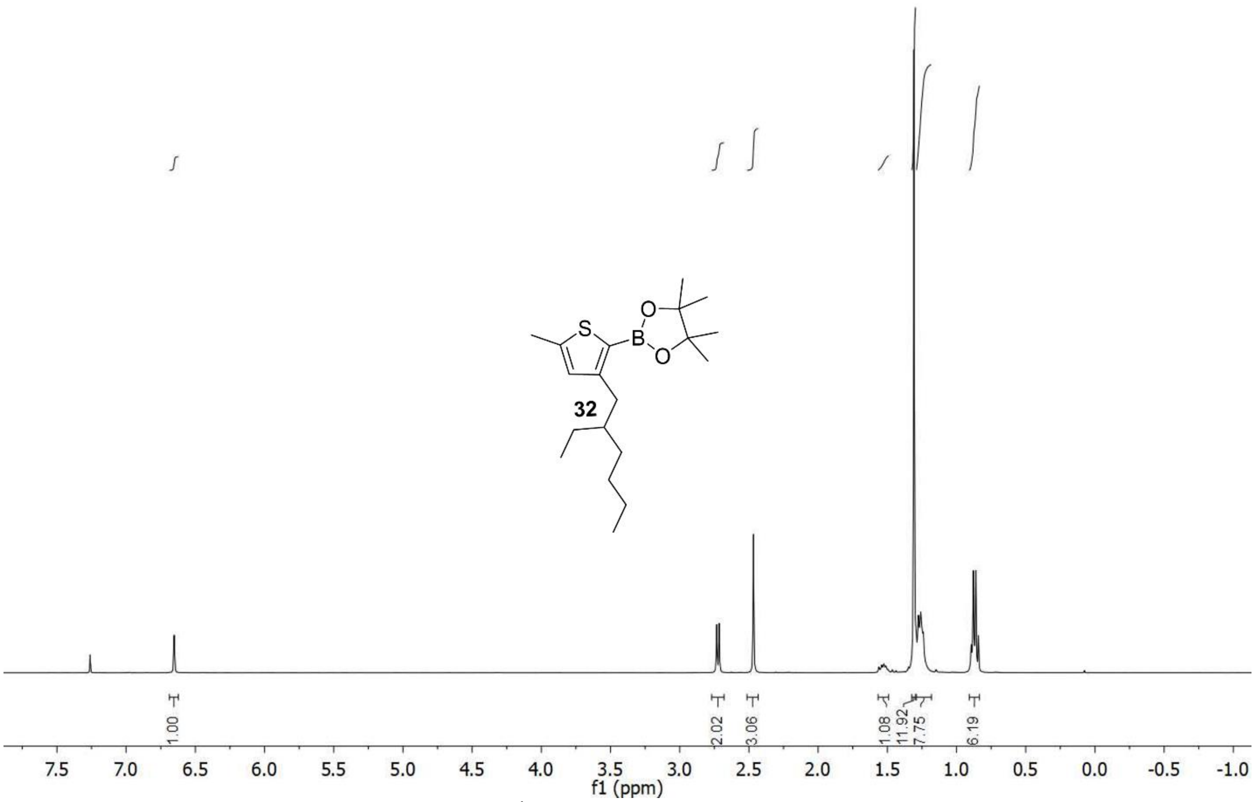

Figure S10: ^{1}H NMR spectrum of **32** in $CDCl_3$.

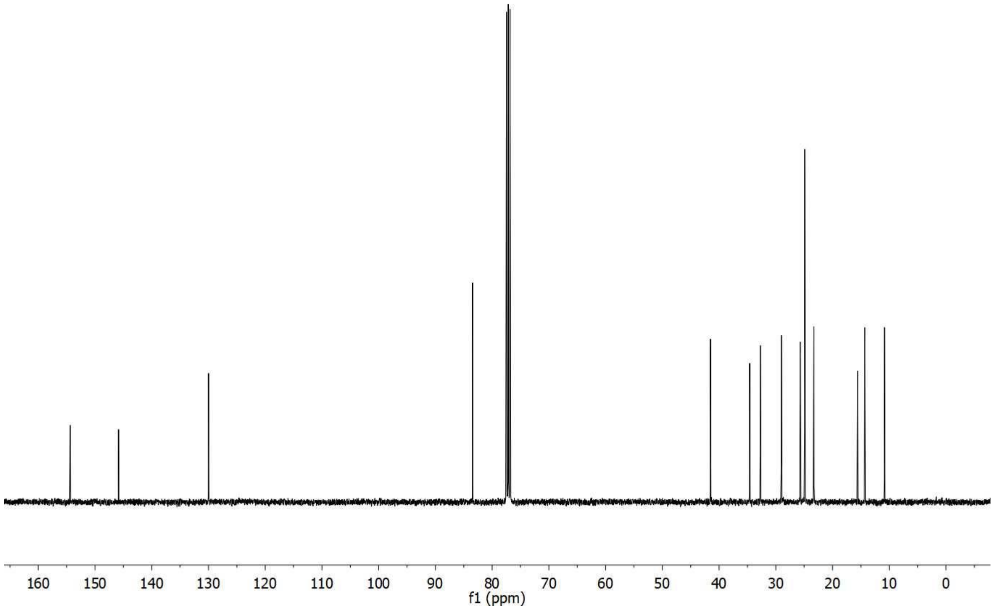

Figure S11: ^{13}C NMR spectrum of **32** in $CDCl_3$.

Intermediate 33

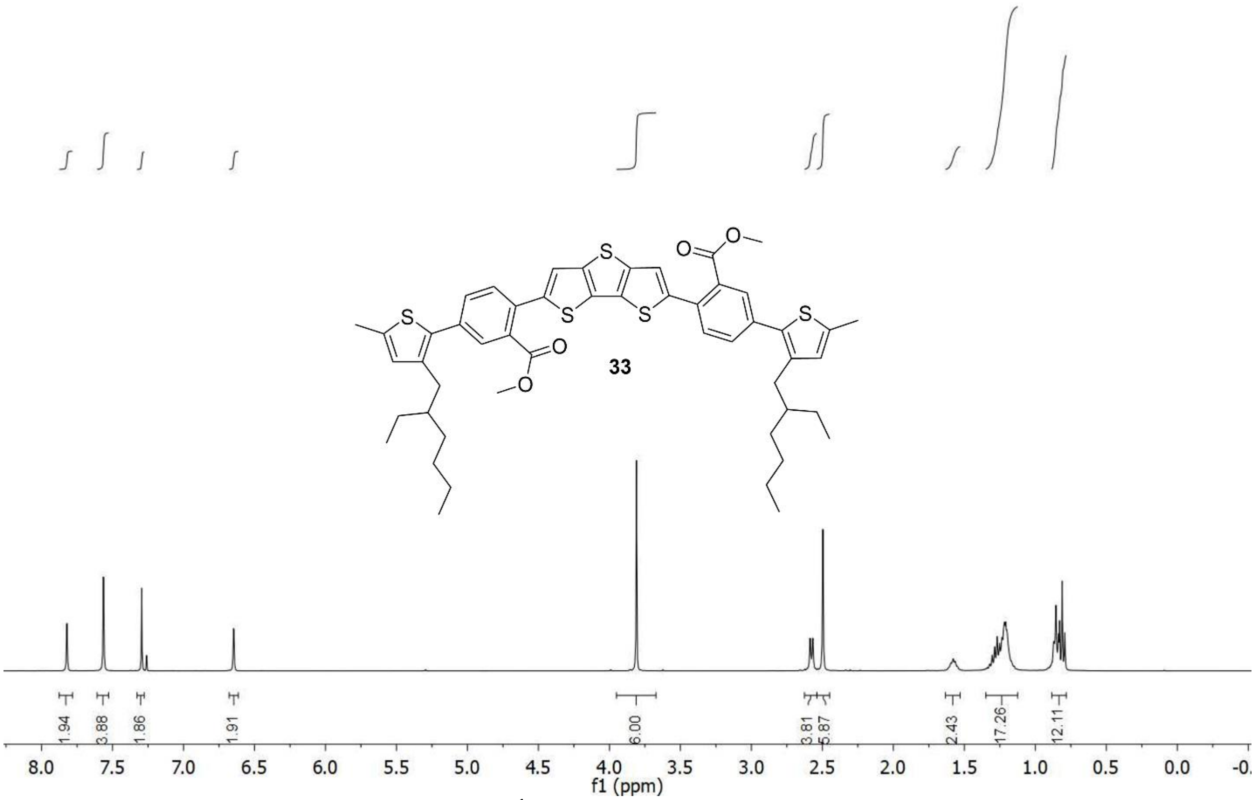

Figure S12: ^{1}H NMR spectrum of **33** in $CDCl_3$.

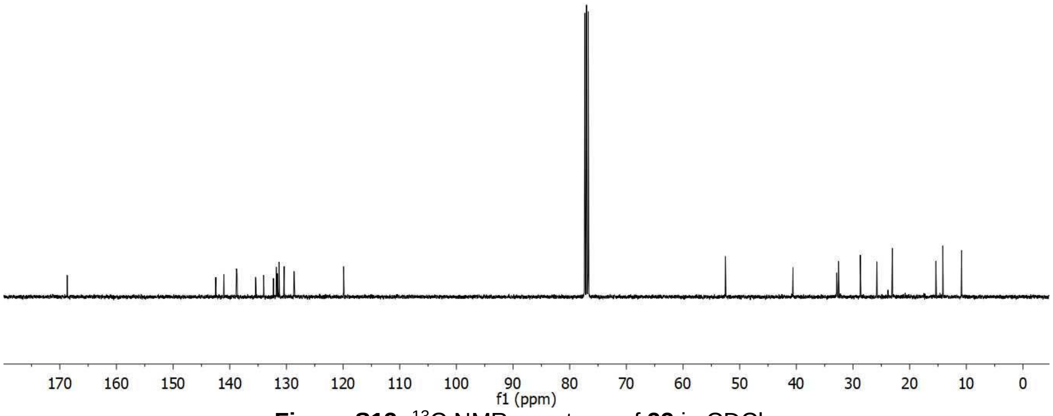

Figure S13: ^{13}C NMR spectrum of **33** in $CDCl_3$.

Intermediate 34

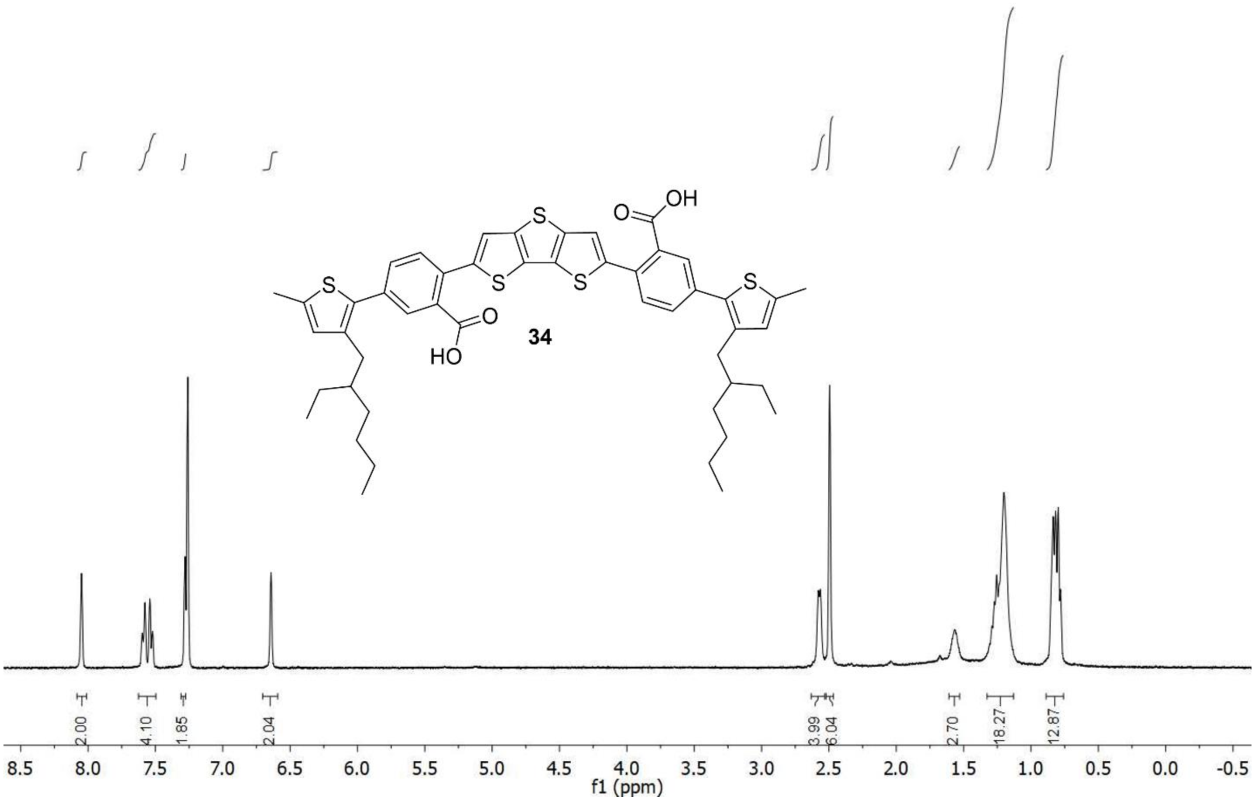

Figure S14: ^{1}H NMR spectrum of **34** in $CDCl_3$.

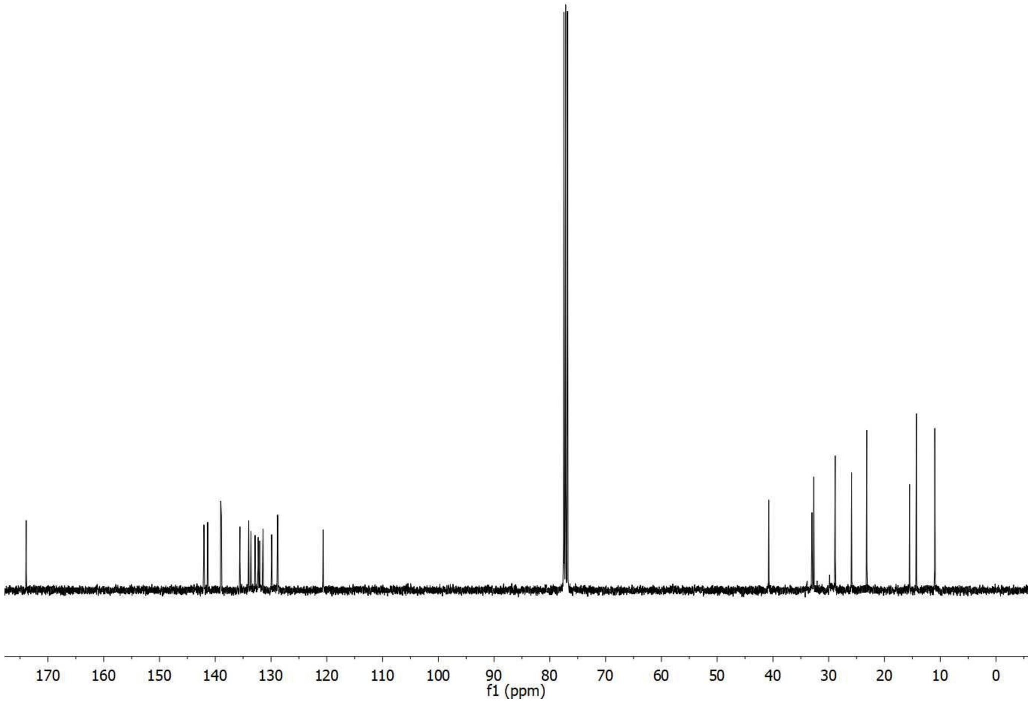

Figure S15: ^{13}C NMR spectrum of **34** in $CDCl_3$.

EtH-T-DI-DTT (1)

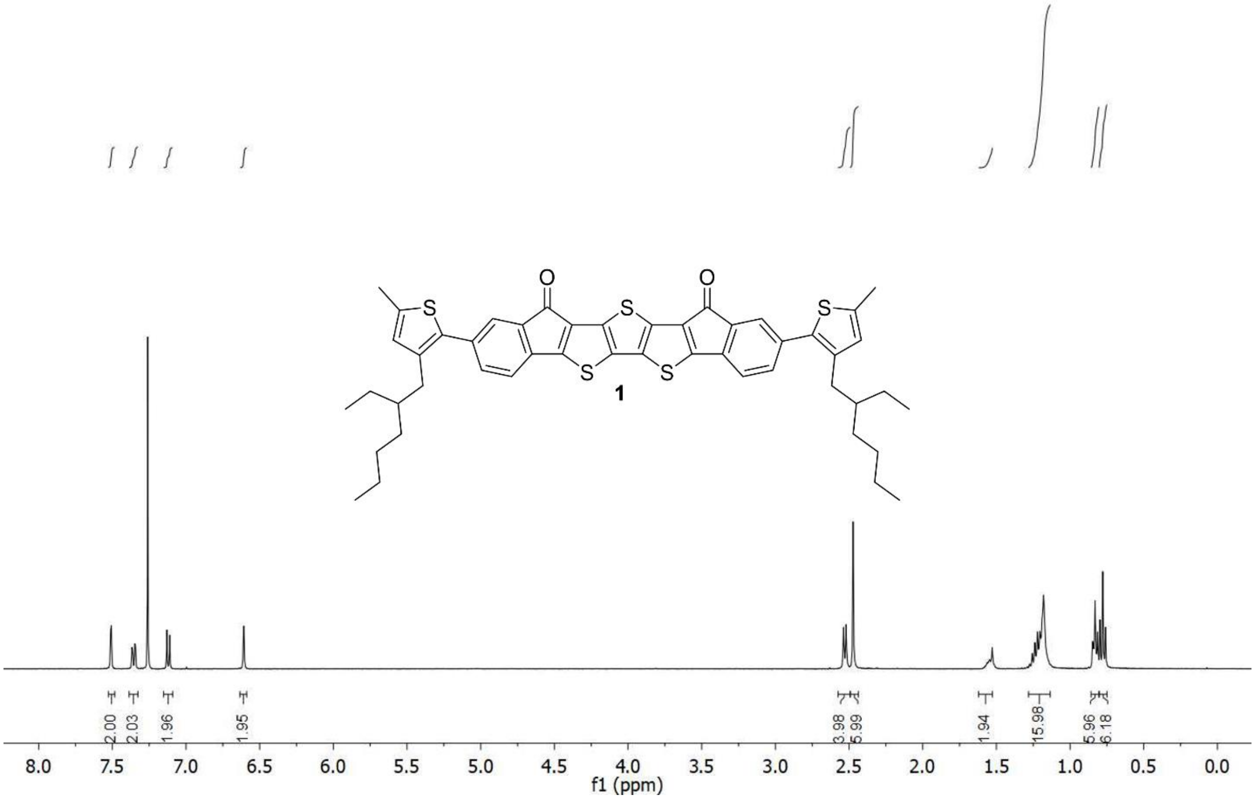

Figure S16: ^{1}H NMR spectrum of **EtH-T-DI-DTT** (**1**) in $CDCl_3$.

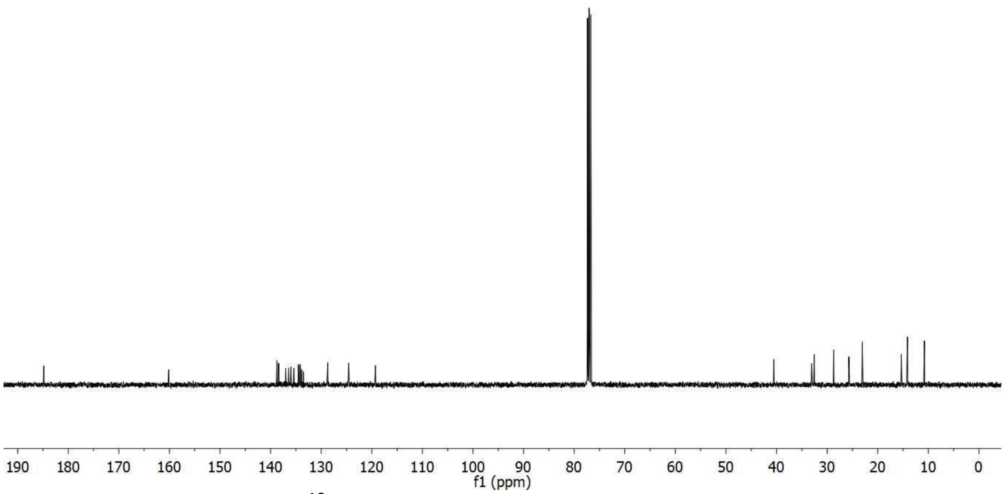

Figure S17: ^{13}C NMR spectrum of **EtH-T-DI-DTT** (**1**) in $CDCl_3$.

4. TGA/DSC

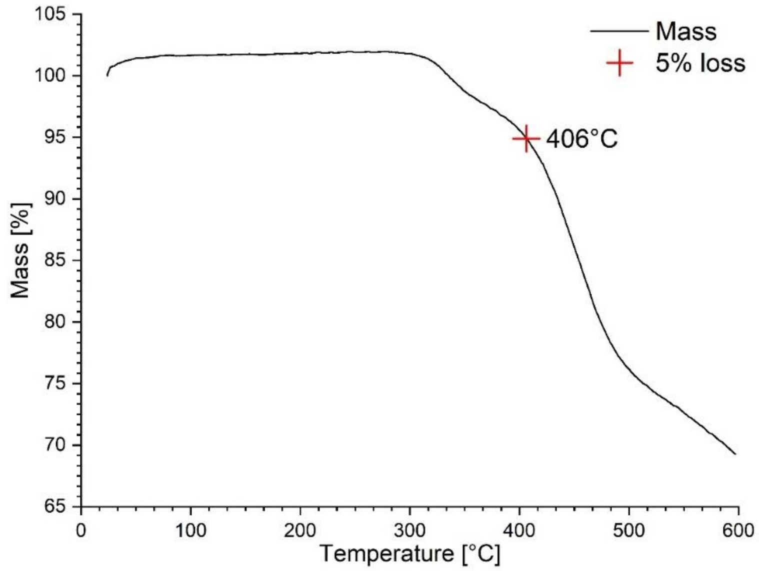

Figure S18: TGA of **EtH-T-DI-DTT**.

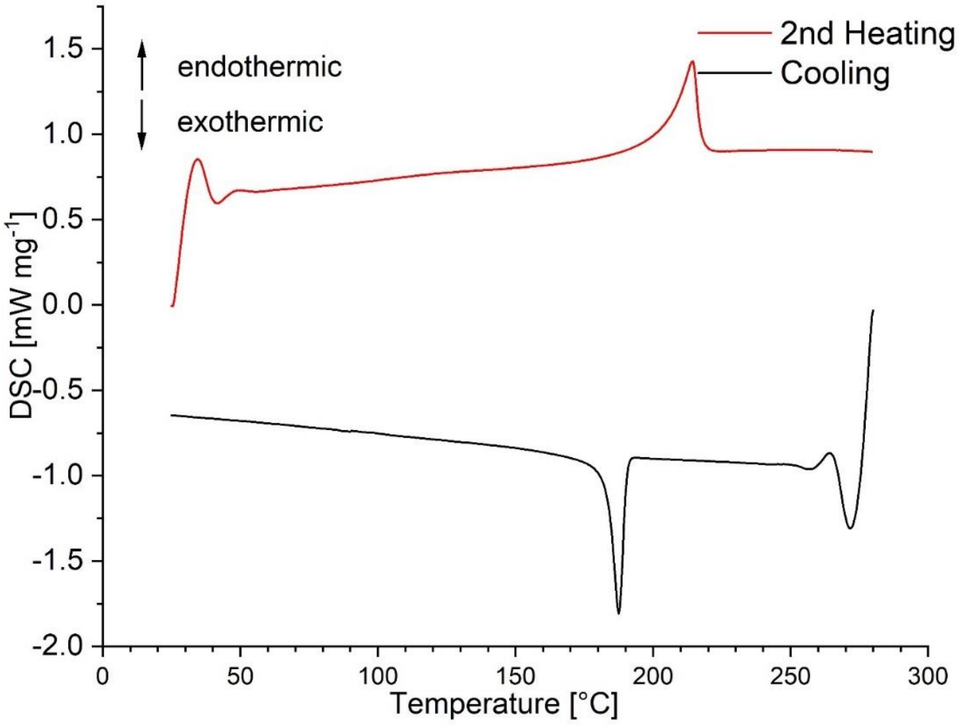

Figure S19: DSC of **EtH-T-DI-DTT**, at a heating rate of 20 K min^{-1}. The signals in the beginning of the heating and cooling cycle are artefacts.

5. UV–vis absorption spectroelectrochemistry

Oxidation process

By increasing the potential gradually from 0 V to +1 V, UV–vis spectroscopy showed an initial absorption profile with λ_{max} at 361 nm and a shallower band at 540 nm. Upon increasing the potential to +1.1 V (the first oxidation peak potential), the high energy absorption band reduced intensity and the low energy band was red-shifted to 550 nm. From +1.3 V (the second oxidation peak) to +1.5 V the low energy absorption extended into the near-IR.

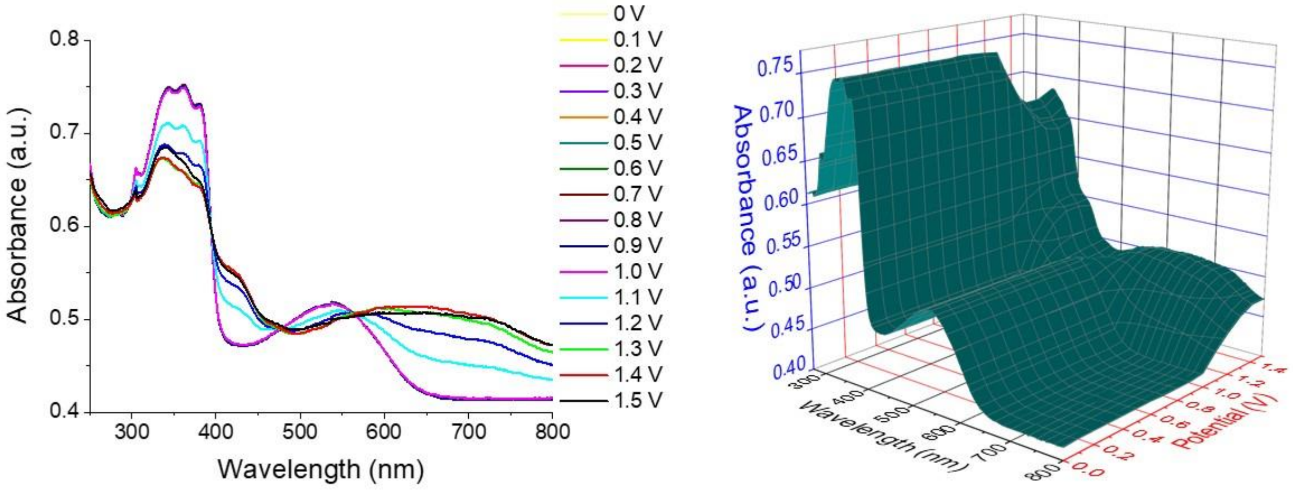

Figure S20: Spectroelectrochemistry plots for **EtH-T-DI-DTT** in solution state for the oxidation process. Conditions: **EtH-T-DI-DTT** *ca.* 0.1 mM was dissolved in DCM with $TBAPF_6$ (0.1 M) as electrolyte, Pt mesh as a working electrode, Pt wire as a counter electrode, and Ag wire as a pseudo reference electrode. Note: The potential is not referenced to Fc/Fc^+ couple.

Reduction process

The reduction processes were observed in the region 0 V to –1.6 V (Figure S21). UV–vis spectra revealed a decrease in intensity with a decrease in the potential with a maximum absorption for the highest energy band at around 341 nm. The low energy band started to disappear at −1.2 V with a new, broader band emerging into the near-IR.

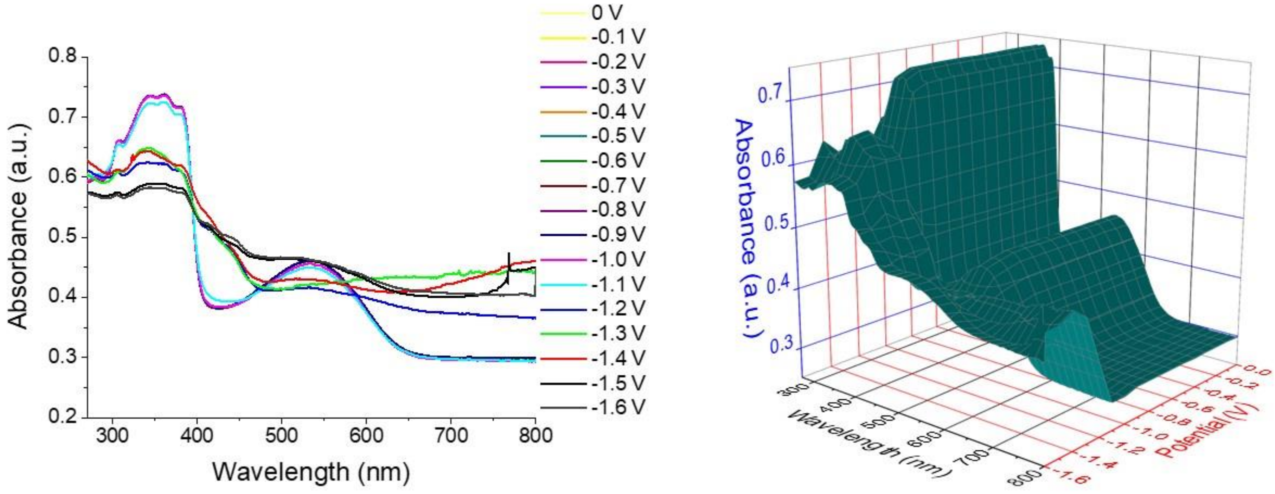

Figure S21: Spectroelectrochemistry plots for **EtH-T-DI-DTT** in solution state for reduction process. Conditions: **EtH-T-DI-DTT** *ca.* 0.1 mM dissolved in DCM with $TBAPF_6$ (0.1 M) as electrolyte, Pt mesh as a working electrode, Pt wire as a counter electrode, and Ag wire as a pseudo reference electrode. Note: The potential is not referenced to Fc/Fc^+ couple.

6. Plots of best performing transistor

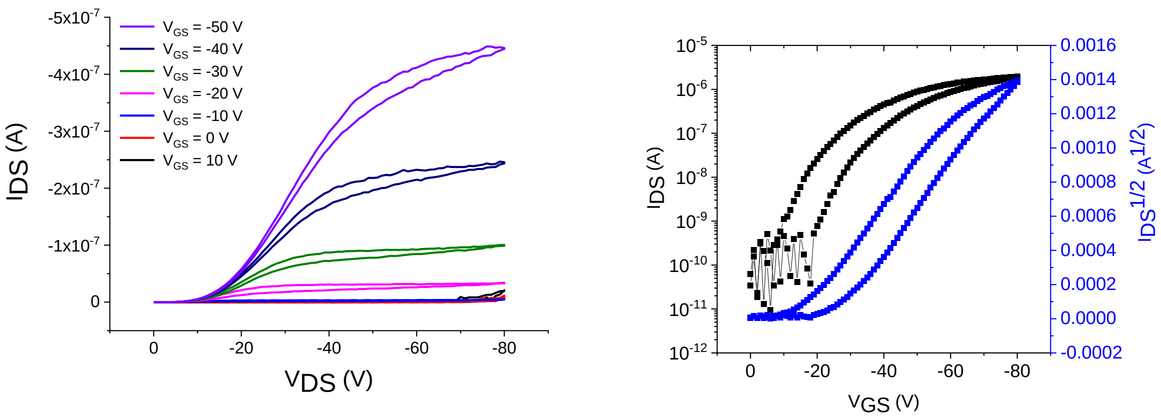

Figure S22: Left: I_{DS}–V_{DS} and I_{DS}–V_{GS} curves (at V_{DS} = −50 V) of the best performing transistor, proceeded with **EtH-T-DTT**, with channel length L = 10 µm, processed with 10 mg mL^{-1} chloroform solution, being spin-coated onto the wafer with 1000 rpm, annealed at 150 °C for 30 min. As SAM, OTS was used.

7. References

1. Guerrero-Sanchez, C.; Keddie, D. J.; Saubern, S.; Chiefari, J. *ACS Comb. Sci.* **2012**, *14* (7), 389-394.
2. Rondeau, R. E. *J. Chem. Eng. Data* **1966**, *11* (1), 124-124.
3. Suwa, Y.; Myint, H. H.; Kurniawan, H.; Ito, F.; Kagawa, K. *Phys. Educ.* **2001**, *36* (4), 293-298.
4. Fulmer, G. R.; Miller, A. J. M.; Sherden, N. H.; Gottlieb, H. E.; Nudelman, A.; Stoltz, B. M.; Bercaw, J. E.; Goldberg, K. I. *Organometallics* **2010**, *29* (9), 2176-2179.
5. Findlay, N. J.; Breig, B.; Forbes, C.; Inigo, A. R.; Kanibolotsky, A. L.; Skabara, P. J. *J. Mater. Chem. C* **2016**, *4* (17), 3774-3780, 10.1039/C5TC03579A.
6. Pommerehne, J.; Vestweber, H.; Guss, W.; Mahrt, R. F.; Bässler, H.; Porsch, M.; Daub, J. *Adv. Mater.* **1995**, *7* (6), 551-554.
7. *Gaussian 09*, Revision D.01; Gaussian, Inc.: Wallingford, CT, 2013..
8. *GaussView*, Version 5.0.9; Gaussian, Inc.: Wallingford, CT, 2008.
9. Kevin, P.; Malik, M. A.; O'Brien, P.; Cameron, J.; Taylor, R. G. D.; Findlay, N. J.; Inigo, A. R.; Skabara, P. J. *J. Mater. Chem. C* **2016**, *4* (22), 5109-5115, 10.1039/C6TC01650B.
10. Shoyama, K.; Mahl, M.; Niyas, M. A.; Ebert, M.; Kachler, V.; Keck, C.; Würthner, F. *J. Org. Chem.* **2020**, *85* (1), 142-149.
11. Coulson, D. R.; Satek, L. C.; Grim, S. O. *Inorg. Synth.* **2007**, *XIII*, 121.
